# The geometry of clinical labs and wellness states from deeply phenotyped humans

**DOI:** 10.1038/s41467-021-23849-8

**Published:** 2021-06-11

**Authors:** Anat Zimmer, Yael Korem, Noa Rappaport, Tomasz Wilmanski, Priyanka Baloni, Kathleen Jade, Max Robinson, Andrew T. Magis, Jennifer Lovejoy, Sean M. Gibbons, Leroy Hood, Nathan D. Price

**Affiliations:** 1 Institute for Systems Biology, Seattle, WA USA; 2 Weizmann Institute, Rehovot, Israel; 3 Providence St Joseph Health, Seattle, WA USA

**Keywords:** Systems biology, Systems analysis, Population screening

## Abstract

Longitudinal multi-omics measurements are highly valuable in studying heterogeneity in health and disease phenotypes. For thousands of people, we have collected longitudinal multi-omics data. To analyze, interpret and visualize this extremely high-dimensional data, we use the Pareto Task Inference (ParTI) method. We find that the clinical labs data fall within a tetrahedron. We then use all other data types to characterize the four archetypes. We find that the tetrahedron comprises three wellness states, defining a wellness triangular plane, and one aberrant health state that captures aspects of commonality in movement away from wellness. We reveal the tradeoffs that shape the data and their hierarchy, and use longitudinal data to observe individual trajectories. We then demonstrate how the movement on the tetrahedron can be used for detecting unexpected trajectories, which might indicate transitions from health to disease and reveal abnormal conditions, even when all individual blood measurements are in the norm.

## Abstract (web summary)

Longitudinal multi-omics measurements are highly valuable in studying heterogeneity in health and disease phenotypes. Here, the authors apply Pareto Task Inference to analyze the clinical lab tests of 3094 individuals and find three wellness states, and one aberrant health state defining this cohort.

## Introduction

To make substantial progress in studying human wellness, there is a need for systematic and holistic approaches that generate and interpret longitudinal health data^1–5^. Emerging technologies allow for thousands of low-cost measurements from individual participants over time^6–9^. Through a partnership with Arivale (a now-closed spin-off company from our lab) we generated such a longitudinal, multi-omics dataset, spanning e.g. genomics, proteomics, metabolomics and microbiome quantification^10–13^. Integrating divergent data types into system-scale analyses represents a major challenge^12,14–16^. Commonly used methods for analyzing high-dimensional data include correlation networks, univariate statistical tests with multiple-hypothesis correction, and multivariate machine learning models^8,9,12–14,17^. These common approaches have successfully been used across various studies. Another approach involves different types of grouping such as clustering and t-SNE^18–22^, and a framework called Multi‐Omics Factor Analysis (MOFA) was suggested to integrate multi-omics data^23,24^. The approach we take herein for dimensionality reduction and analyzing broad features of the high-dimensional data is Pareto Task Inference (ParTI)^25^. This approach is based on an evolutionary theory and its main concept is that if data points of a high-dimensional dataset fall on a simple shape like a line, a triangle, a tetrahedron, it is due to tradeoffs in the biological system, rather than by chance^25,26^. This method also computes statistical significance for the resulting simplex^25^. If a significant simplex is found, the vertices are denoted archetypes, that specialize in a certain task, with tradeoffs among these tasks. Enrichment analysis of any measurable feature can be used to characterize the archetypes and uncover the tradeoffs. The ParTI method has several advantages: it allows the analysis of a dataset as a continuous space rather than deterministic grouping, it does not require prior knowledge for characterizing the archetypes and revealing the tradeoffs, and due to the geometric representation of the data - the visualization of a high-dimensional dataset and the overlay of different data types is straightforward^25–27^. Multiple studies have successfully used this method to analyze different types of high-dimensional data, such as tumor mRNA expression data^28^, and single-cell data^29,30^. Here, we apply the ParTI method to analyze the high dimensional dataset of personalized data clouds obtained by Arivale. We find that the clinical lab data-points fall on a significant tetrahedron. We then use all other data types to characterize the phenotypic features of the four archetypes and reveal the fundamental tradeoffs that define these states. We find that both the discrete (questionnaire data) and the continuous variables (the four ‘omics’ data types) indicate three wellness states and one aberrant health state. We then show how longitudinal data and the movement on the tetrahedron can be used for early detection of transitions from health to disease state, and for identifying abnormal conditions.

## Results

### The Arivale cohort

Participants provided blood and stool samples every six months, filled out questionnaires about their health history and lifestyle habits, and used a Fitbit activity tracker. From the blood samples, 124 clinical lab tests, 990 metabolites and 256 proteins were measured (see Methods), and the DNA was sequenced (whole genome sequencing for 2876 and SNPchip for 1948). Based on these measurements and the participants goals, health coaches guided the participants on how to change their lifestyle to optimize their health. The program was available for 5 years and included nearly 5000 participants that had between 1 to 8 timepoint^10,11^. Most participants in the Arivale wellness program consented for their deidentified data to be used for research purposes, which are analyzed herein.

### Clinical labs fall on a significant and robust tetrahedron

**Fig. 1:**
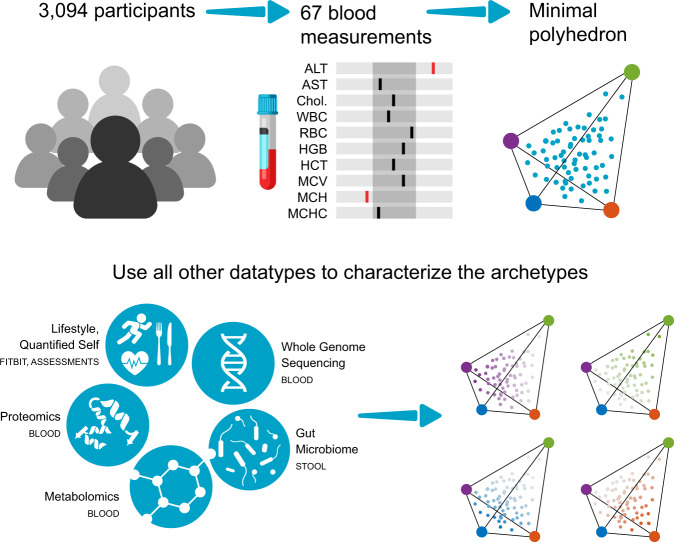
Pareto task inference of clinical labs—study overview. 67 blood measurements from 3094 individuals were used for the Pareto task inference analysis to find the minimal significant polyhedron and the position of the archetypes (the vertices). After finding the polyhedron using the clinical labs, all other data types (lifestyle self-administered questionnaires and Fitbit records, genomics, microbiome, metabolomics and proteomics) were used to find enriched traits close to every archetype in order to characterize the archetypes and reveal the tradeoffs in the system.

**Fig. 2:**
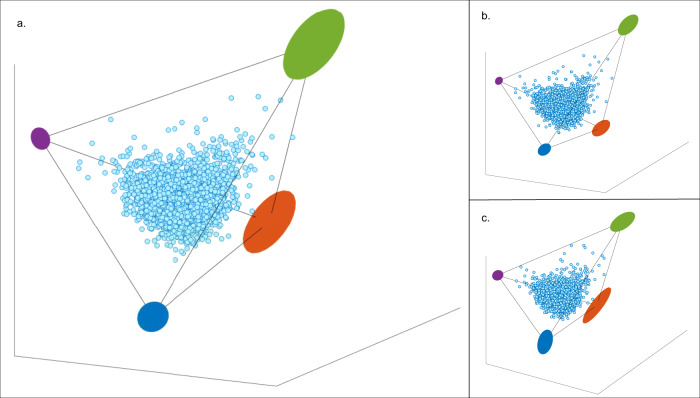
The clinical labs dataset falls on a significant tetrahedron (*t*-test *P*-value < 0.001). **a** The dataset is composed of *n* = 3094 participants and 67 blood measurements, displayed on the first 3 PCs space (light blue dots). The colored ellipses designate the archetypes’ possible positions with error after 1000 times of bootstrapping. **b**, **c** The tetrahedron is robust to data selection. The participants in the cohort have multiple visits (between 1–8). To test the robustness of the tetrahedron, we randomly selected one visit per participant and constructed different data sets. Then, for every data selection we ran the ParTI analysis and found that for different data selections we receive significant tetrahedrons (*t*-test *P*-value <0.05). Out of 7 data selections 4 were significant, 3 runs had a *t*-test *P*-value < 0.001 (two of them are shown here in **b** and **c**), and the rest had a *t*-test *P*-value=0.04, 0.06, 0.07, 0.28 (see Methods).

Following data cleaning and normalization (see Methods) we retained 67 clinical lab analytes (Supplementary Dataset 1), and 3094 individuals. 42% of the individuals were male and 58% were female (Supplementary Fig. 1). Age was normally distributed for both males and females (Supplementary Fig. 2), with a mean age of 48.4 (±12.5) for men and 48.8 (±12.2) for women. We then applied the ParTI analysis and found that the clinical labs dataset falls within a significant tetrahedron (*P*-value < 0.001, Figs. 1, 2). We applied the method with a different number of archetypes (*n* = 2.3), however, we did not get a significant *P*-value (*P*-value = 0.51, 0.502 respectively). For 5 archetypes we received a *P*-value = 0.001, which was not significant after correcting for multiple hypothesis testing. We applied the analysis with various types of data sampling repeatedly resulting in significant *P*-values, showing that the tetrahedron is robust to data selection (see Methods, and Fig. 2).

### Characterizing the four archetypes using enrichment analysis with all other data types

The clinical lab matrix was used to construct the tetrahedron, and we used all other data-sets (20 matrices, 12,848 variables) to characterize the archetypes and reveal biological trade-offs (Fig. 1). We applied the enrichment analysis as described in Hart et al. (see Methods)^25^. In short, we were looking for features that are maximized close to an archetype and decay as they move away from the archetype to any direction. We tested for enrichment of all the variables at each archetype using all data-points in every test (see Methods, Fig. 1). We used the Bonferroni correction to correct for multiple hypothesis testing. The full table of features and *P*-values can be found in Supplementary Dataset 2.

### Enrichment analysis of the self-reported assessments indicates four distinct health states

**Fig. 3:**
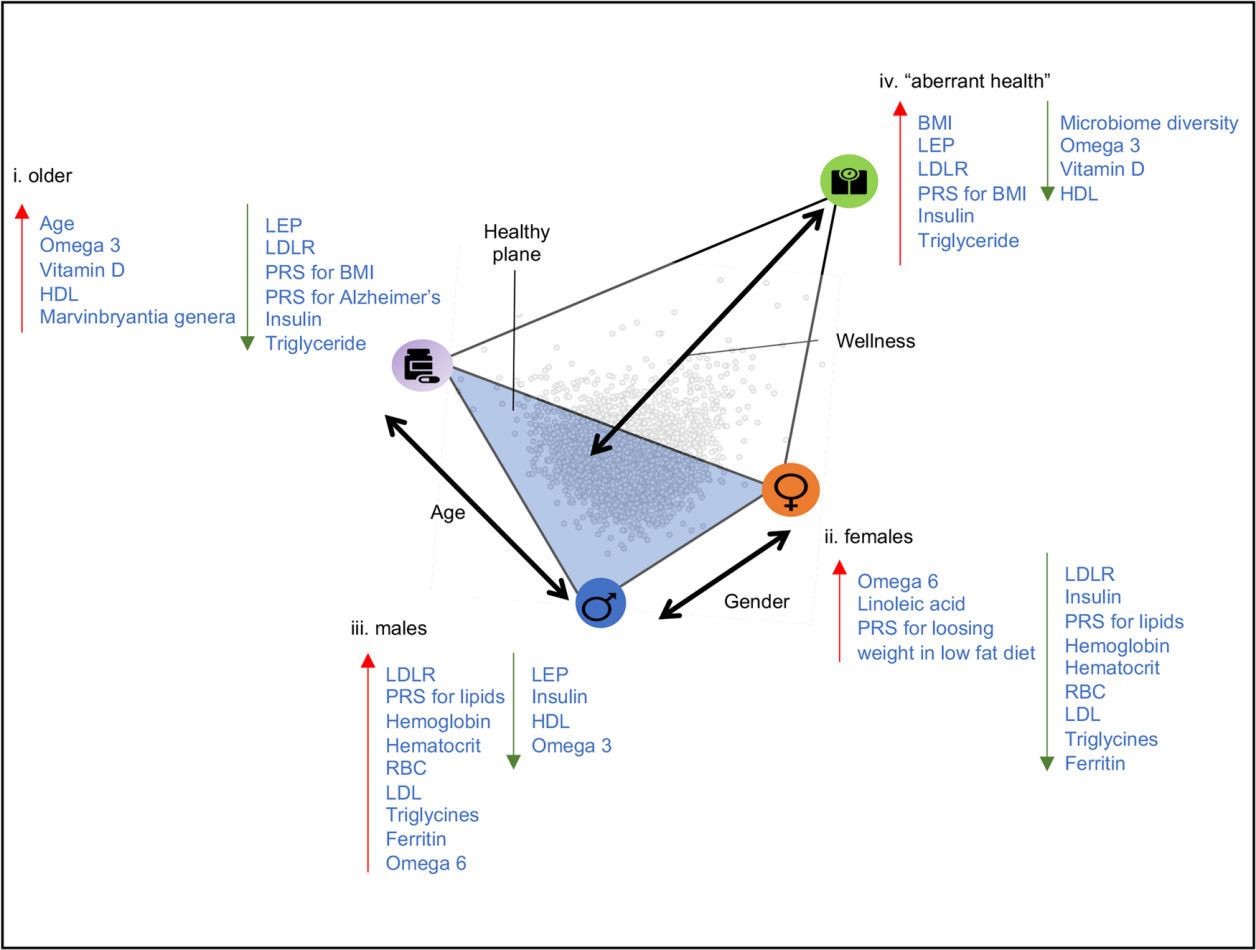
The enrichment analysis revealed one aberrant health state, and three wellness states: (1) the older-archetype (2) the female-archetype (3) the male-archetype. Written in blue are the traits that are found to be enriched close to every archetype at high (left) or low (right) levels. Also shown are the major axes of the data variation (also shown and described in Fig. 4, *n* = 3094).

We first describe the discrete variables (demographic, clinical information, and the self-reports), which are textual categories that are easy to interpret and provide a brief characterization of every archetype (Table 1). Archetype I was enriched for older ages (mean of 57 in the first bin close to the archetype vs mean of 48 for the rest of the data), for having a partner and grandchildren (*P*-value = 2e−07, 6.9e−07, Table 1), but was not enriched for gender (Supplementary Dataset 5). According to the self-reports, Archetype I was enriched for taking supplements, eating cruciferous vegetables daily, experiencing satisfaction from life and being physically active (Table 1). Archetype II was enriched for females (Supplementary Dataset 5), vegetarian diet and an active life-style. Although this archetype was enriched for happiness and satisfaction in general, it was also enriched for changes in mood, and experiencing stress (Table 1). Archetype III was enriched for males (Supplementary Dataset 5), for not eating fruits or breakfast, for consuming alcoholic drinks daily with a preference for beer, and for good physical and mental feeling. This archetype was also enriched for non-responders (individuals who did not respond to a particular question). Archetype IV was not enriched for gender (Supplementary Dataset 5) or age, but was enriched for high BMI and high weight. It was enriched for not drinking alcoholic drinks, and for drinking sugary drinks. Participants adjacent to this vertex were more likely to report aberrant health (diarrhea, reflux, etc.), high appetite and diabetic diet. They were not satisfied with their appearance and their physical and mental condition (Table 1). From this analysis we conclude that the bottom triangle of the tetrahedron comprises three “healthy” archetypes – I. the older archetype II. the female-archetype, and III. the male-archetype. Archetype IV, at the far edge of the tetrahedron (mean Euclidean distance between archetypes I,II,III is 29.8 (±2.6), and the mean distance between the lower triangle to Archetype IV is 38.5 (±2.8)), is markedly different from the first three, and in general is enriched for traits associated with poor health- the unhealthy-archetype (Table 1, Fig. 3). Importantly, these traits were self-reported and found to be enriched close to the archetypes, and that the data points were not clustered or grouped together based on these traits (such as age and gender). The spatial organization of the data points was determined by the blood chemistry profiles and plotted in 3D using dimensionality reduction (PCA) for visualization purposes (see Methods, Fig. 2). Additionally, if for example an archetype is enriched for males, it means that close to that archetype there is a higher rate of males compared to random sampling (97% are males in the first bin). Next to archetype II that was enriched for females, 100% of the individuals in the first bin were females (Supplementary Dataset 5).

**Table 1:** Enrichment analysis of demographic features and self-reported questionnaires reveals the archetype characteristics.

		Archetype 1			Archetype 2			Archetype 3			Archetype 4		
		Mean first	Mean rest	*P*-value	Mean first	Mean rest	*P*-value	Mean first	Mean rest	*P*-value	Mean first	Mean rest	*P*-value
Continuous features	Age	58	48	**2.6E−19**	40	49	**8.9E−18**	45	49	3.0E−05	49	49	8.3E−01
	BMI	25	28	**4.4E−08**	25	28	**4.4E−10**	28	27	5.9E−06	37	27	**1.6E−56**
	Weight	164	180	3.6E−05	152	180	**2.7E−19**	202	178	**1.5E−18**	241	176	**9.4E−49**
	Mean arterial blood pressure	98	92	**6.6E−11**	86	92	**9.1E−13**	91	92	2.8E−01	102	91	**3.0E−23**
	Systolic	125	125	8.0E−01	115	126	**6.5E−16**	130	125	**3.2E−06**	139	124	**4.1E−26**
	Diastolic	74	76	3.0E−02	71	76	**2.0E−08**	81	75	**8.3E−12**	83	75	**4.0E−17**
		**Feature name**		***P*-value**	**Feature name**		***P*-value**	**Feature name**		***P*-value**	**Feature name**		***P*-value**
Discrete features	Gender	—		—	sex: F		**0.0E + 00**	sex: M		**0.0E + 00**	—		—
	Familial status	Live with: (2) Spouse/partner		**2.0E−07**	Relationship status: (1) Partnered		1.7E−04	—		—	—		—
		grandchildren: (2) Two		**6.9E−07**	—		—		—	—	—		—
	Diet	cruciferous vegetables: (0) Daily		3.4E−04	Lifestyle diet: (1) Vegetarian		1.1E−03	fruits: (0) Zero/less than 1 per day		**1.9E−07**	diet: (7) Diabetic		**8.3E−11**
	Alcohol consumption	alcohol days a week: (4) Daily		4.0E−05	—		—	alcohol type: (1) Beer		**6.3E−07**	alcohol days a week: (0) I do not drink		2.2E−05
		alcohol type: (2) Wine		7.9E−03	—		—	alcohol drinks a day: (2) 3-4 drinks		1.4E−04	alcohol type: (3) Liquor		1.1E−03
	Medication	medications: (no response)		**1.5E−11**	medications: (4) Not at all		2.7E-03	medications: (2) Several times per week		1.9E−02	medications: (1) Daily		**4.1E−07**
	Supplement uptake	supplements: (1) Daily		4.2E−03	supplements: (3) Once per week or less		2.8E−03	supplements: (4) Not at all		8.5E−03	supplements: (4) Not at all		3.6E−04
	Activity	moderate activity: (0) At least 7 times per week		4.5E−05	moderate activity: (4) Less than once per week		3.9E−03	moderate activity duration: (2) 20 min		1.2E−02	vigorous activity: (5) Rarely or never		**7.6E−12**
		vigorous activity duration: (2) 20 min		3.5E−03	vigorous activity duration: (3) 30 min		4.9E−02	vigorous activity: (2) At least 3 times a week		4.8E−02	time seated: (0) Most of the time (work + relaxing 12 hours)		**7.5E−10**
	Personality	easily disturbed: (4) Disagree		2.1E−03	stressed easily: (1) Strongly agree		2.8E−04	life of party: (4) Agree		2.1E−04	easily disturbed: (1) Strongly agree		2.6E−02
		worry: (3) Neither disagree nor agree		7.0E−03	feel others emotions: (5) Strongly agree		4.3E−04	relaxed: (5) Strongly agree		1.0E−05	do chores: (1) Strongly disagree		4.3E−05
		do chores: (4) Agree		1.4E−03	—		—	—		—	forget toֿ put things in proper place: (2) Agree		3.9E−04
	General feeling	not healthy: (6) Strongly Disagree		**1.7E−06**	—		—	—		—	not healthy: (1) Strongly Agree		**3.6E−07**
		healthy life: (5) Strongly agree		**1.6E−06**	—		—	—		—	have energy: (1) Strongly Disagree		**8.3E−12**

(1) Healthy and older population of the cohort. (2) Healthy and relatively young females. (3) Healthy and relatively young males. (4) Aberrant health—enriched for high BMI, high blood pressure, not enriched for gender specific or age. Selected continuous features are shown in the upper part of the table. “Mean first” stands for the mean value in the first bin closest to the archetype, “Mean rest” stands for the mean value of the rest of the data. Selected discrete features are shown in the lower table. Different features from the same category were found to be enriched closest to the archetypes. The blank cells represent no enriched features. Highlighted *P*-values< 3.4e−06 which are significant after Bonferroni correction. The full table of features per archetype and their *P*-value can be found in Supplementary Dataset 2.

### Enrichment analysis of polygenic risk scores (PRS)

Next, we analyzed the enrichment of the polygenic risk scores (PRS), designating a continuous measure of risk aggregating the effects of multiple SNPs. We found that the PRS for high BMI was increased adjacent to the unhealthy-archetype (*P*-value = 4.8e−11), this archetype was also enriched for high BMI. Other traits were enriched close to the different archetypes, but their *P*-values did not pass the Bonferroni correction threshold, these include: high risk for high LDL, triglycerides, and HDL close to the male-archetype (*P*-value = 2.5e−03, 2.7e−02, 3.5e−02). The female-archetype was enriched for lower PRS for HDL and LDL (*P*-value = 2.9e−03, and 1.9e−02), and high PRS for losing weight from a low-fat diet (*P*-value = 2.3e−04). The older-archetype was enriched for low PRS of BMI and Alzheimer’s disease (*P*-value = 1.72e−05, and 2.4e−02 respectively, Fig. 3).

### Microbiome enrichment analysis

Shannon index, observed species, and Chao1 are metrics that indicate the diversity of the gut microbiome, and higher diversity is often associated with better health^13,29,31–33^. We found that all these three metrics were low near the unhealthy-archetype. Other archetypes did not show any significant relationship with microbiome diversity (Fig. 3). Thirty gut bacterial genera were significantly enriched in particular archetypes after Bonferroni correction (*P*-value < 3.89e−06). 29 of these genera were enriched next to the unhealthy-archetype, and 26 of the 29 showed lower abundance next to that archetype (a depletion of 26% of the genera that were tested). Depletion of bacteria species and low diversity are associated with many disease conditions^13,17,31,34^. Among the depleted genera are *Faecalibacterium*, *Ruminococcaceae UCG-005, Christensenellaceae R-7* group and *Lachnospiraceae*, which have been associated with a healthy gut ecosystem^35,36^ through the fermentation of dietary fiber and the production of butyrate and other short-chain fatty acids. Three genera showed significant enrichment with high levels in archetype IV: *Bacteroides, Lachnoclostridium* and *Megasphaera*. *Bacteroides* is one of the most common genera in the gut microbiome, and an increase of this genus is associated with several conditions like inflammation, type 1 diabetes, and severe diarrhea^37–42^. *Lachnoclostridium* showed an increase in abundance following cefprozil treatment (antibiotic)^43^, and *Megasphaera* was enriched in obese compared to lean twins^44,45^. The *Marvinbryantia* genus was significantly enriched in the older-archetype (Fig. 3). Increase in *Marvinbryantia* was linked with lowering blood pressure in a rat model of hypertension^46^.

### Metabolomics enrichment analysis

A total of 990 plasma metabolites were measured for every participant, of which 45% were lipids, 18% Amino Acids, 8% Xenobiotics, and 18% unknown (Supplementary Fig. 3). The four archetypes had distinctive signatures of enriched metabolites (Supplementary Fig. 4), with no common metabolites that were shared in all the four archetypes. The unhealthy-archetype was the most metabolically perturbed with 216 enriched metabolites. Some of the most enriched metabolites were lipids containing saturated fatty acids (palmitic and stearic), indicative of a poor diet. On the contrary, the older-archetype was enriched for a variety of omega-3 fatty acids containing lipid species, while depleted in lipid species containing saturated fat. 9/16 (56%) of the depleted metabolites next to the older-archetype were omega-6 fatty acid containing lipids (arachidonic and adrenic acids), which were at high abundance close to the unhealthy-archetype. These findings indicate better dietary habits and/or supplement use in individuals close to the older-archetype. The male-archetype had 56 enriched metabolites, of which 10 overlapped with the unhealthy-archetype (the biggest overlap). The female and male archetypes showed a tradeoff between the sex hormones, where the androsterones—the precursor for testosterone, androsterone glucuronide and DHEA-S were enriched in the male-archetype, and pregnenediol was enriched in the female-archetype. Other sex related metabolites such as creatinine, which correlates with lean muscle mass and tends to be higher in males than females^47^, showed a similar trend. Only eight other metabolites were enriched in the female-archetype, five of them were Plasmalogens. Plasmalogens are found in various human tissues, especially in the nervous, immune, and cardiovascular system, and have a role in signal transduction, membrane dynamics and in protecting cells from reactive oxygen species damage^48^. All eight metabolites that were enriched in the female-archetype, were depleted from the unhealthy-archetype. Branched chain amino acids (BCAA) leucine, valine, and isoleucine, were depleted in the female-archetype, and enriched in the male-archetype. This tradeoff might indicate differences in diet, since BCAAs are high in animal products such as meats and eggs, while plant-based diets are generally characterized by lower BCAA content^49^. BCAAs were also enriched in the unhealthy-archetype. Consistent with this enrichment pattern, elevated circulating BCAA levels have been previously associated with increased risk of cardiovascular and metabolic diseases^50,51^. The shared BCAA metabolic signatures among the male- and unhealthy-archetypes highlight potential similarities between these two archetypes, based on similar dietary habits, and possible shared physiological perturbations (Supplementary Dataset 2).

### Proteomics enrichment analysis

A total of 265 plasma proteins were measured from two Cardiovascular Disease (CVD) panels, and one inflammatory panel. Our analysis identified 136 proteins enriched in the unhealthy-archetype, 16 in the male-archetype, 38 in the female-archetype, and only seven for the older-archetype. Only 11 proteins were found at significantly lower levels in the unhealthy-archetype, while 125/136 (92%) were at significantly higher levels. Among the 11 less abundant proteins was Paraoxonase 3 (PON3), which is associated with HDL levels. The most significantly low abundant protein for the older-archetype was leptin (LEP) (*P*-value = 4.3e−11). LEP was depleted also in the male-archetype, and enriched in the unhealthy-archetype (Fig. 3). Low-density lipoprotein receptor (LDLR) was in lower abundance adjacent to the older and the female archetypes, and more abundant adjecent to the males and the unhealthy-archetypes (Fig. 3). These findings are consistent with the high LDL levels observed in the clinical labs (Fig. 3), and the metabolomics. Only 2/37 proteins that were enriched close to the female archetype had significantly higher levels.

### The clinical labs profiles at the archetypes reveal the main axes of variation and their order: (1) the wellness axis, (2) the age axis, (3) the gender axis

**Fig. 4:**
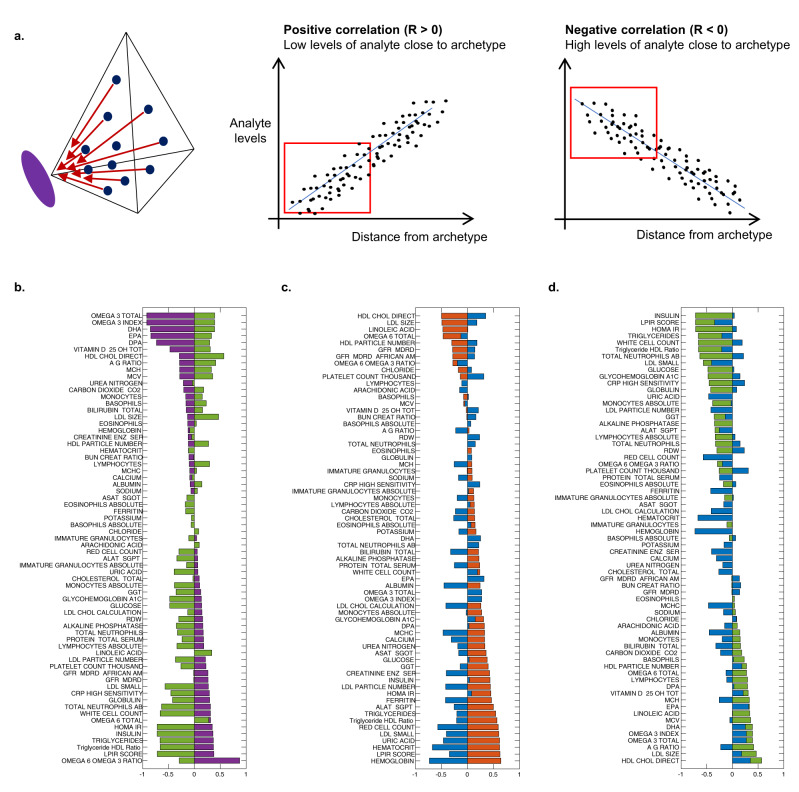
Correlations between the distances from the archetypes and analyte levels reveal the principal analytes next to every archetype, and the trade-offs in the data. **a** Schematic view: the distances between the data-points and the archetypes were correlated with the levels of each analyte. Positive correlation (*R* > 0) means that low levels of the analyte correlate with shorter distance from the archetype, negative correlation (*R* < 0) means that higher levels of the analyte correlate with shorter distances from the archetype. **b** The correlation coefficients of analyte levels with distance from Archetype I were ordered and presented in the horizontal bar plot (purple), and compared to the correlation coefficients of the analytes with distances from Archetype IV (green bars). **c** The same as **b** for Archetype II (orange bars) and Archetype III (blue bars), analytes were ordered according to the correlation coefficients of Archetype II. **d** The same as **b** for Archetype IV (green bars) and Archetype III (blue bars), analytes were ordered according to the correlation coefficients of Archetype IV. The full table of the correlation coefficients can be found in Supplementary Figs. 5–7.

To better understand which analytes from the blood-chemistries were the most influential in determining the spatial position of the data points, we calculated the correlation between the distances of the data-points to an archetype and the analyte values for every archetype and every analyte, and ranked the analytes according to the correlation coefficients in descending order (Supplementary Figs. 5–7). We found that high levels of omega-3 total, DHA, DPA and vitamin-D are correlated with shorter distances to the older-archetype, which is consistent with the enrichment of the self-reported supplements uptake, since the richest sources of these nutrients are derived from dietary supplementation. We found that low levels of triglycerides, insulin, LDL cholesterol, white blood cell count, and lower insulin resistance scores (lipoprotein insulin resistance (LPIR) and HOMA-IR), were correlated with greater distances to this archetype, which supports the conclusion that this archetype is characterized by better health. In contrast, high levels of triglycerides, insulin, LDL cholesterol, and WBC, and higher LPIR and HOMA-IR scores, were correlated with proximity to the unhealthy-archetype. This reciprocal image of the correlations between the older archetype and the unhealthy-archetype nicely demonstrated the trade-offs between health and aberrant health states according to the clinical labs (Fig. 4, Supplementary Fig. 7). Another trade-off can be seen between the male and female archetypes, with positive correlation between hemoglobin, hematocrit, red cell count and creatinine with distances from the female-archetype, and anti-correlation of these markers and the distances from the male-archetype (Fig. 4). High levels of LDL were correlated with shorter distances to the male-archetype, which is consistent with high PRS for high levels of lipids, and high levels of lipids measured in the metabolomics dataset described earlier. However, insulin, glucose, HOMA-IR, CRP, and white cell count correlated with greater distances to this archetype, suggesting that this archetype is also characterized with healthy individuals in opposed to the unhealthy-archetype (Fig. 4).

**Fig. 5:**
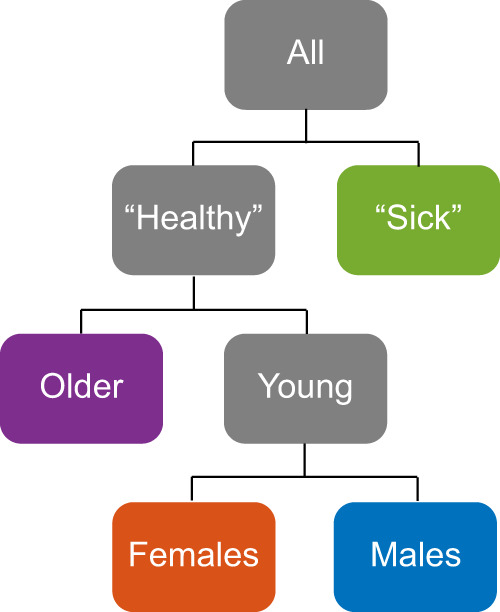
The hierarchy of the principles that shape the spatial organization of the data-points. The first PC is correlated to markers of disease state like LPIR, insulin, HOMA IR (diabetes), triglyceride, LDL, (Cardio-vascular disease), white cell count, neutrophils (inflammation). And therefore, the first split of the data is according to wellness state. The second PC is correlated with omega 3 in different forms. High levels of omega 3 are achieved from supplement uptake, which characterize older individuals and therefore the second split is based on supplement uptake / or age. The third and 4^th^ PC’s are correlated with lipids and markers like: Hemoglobin, Hematocrit, Red cell count, that separate the males and females and therefore the last split is according to sex (Table 2).

Another way to reveal the most impactful analytes that determined the spatial spread of the data points is by correlating the analytes with the PC coefficients (Table 2). We found that the first PC was highly correlated (*R* > 0.5) with diabetes markers such as insulin, LPIR, HOMA IR, and cardiovascular diseases markers such as triglycerides, and LDL, and anti-correlated (*R* < −0.5) with HDL and Adiponectin serum. These results suggest that the first axis separates the data based on health status (“healthy” versus “unhealthy”), that is reflected in the clinical labs (Table 2, Fig. 5). Diseases that are not reflected in the clinical labs, like mental-illnesses for instance, are most likely not captured by this analysis.

**Table 2:** Correlation between the PCs and the analytes reveals the dominant analytes that shape the data and demonstrate their hierarchy.

PC 1		PC 2		PC 3		PC 4	
**LPIR SCORE**	**0.78**	**OMEGA 6 OMEGA 3 RATIO**	**0.70**	EPA	0.47	**CHOLESTEROL TOTAL**	**0.70**
**Triglyceride HDL Ratio**	**0.71**	OMEGA 6 TOTAL	0.45	OMEGA 3 TOTAL	0.45	**LDL CHOL CALCULATION**	**0.65**
**TRIGLYCERIDES**	**0.71**	LINOLEIC ACID	0.39	OMEGA 3 INDEX	0.45	**LDL PARTICLE NUMBER**	**0.60**
**INSULIN**	**0.67**	GFR MDRD	0.37	TOTAL NEUTROPHILS AB	0.41	**LYMPHOCYTES**	**0.51**
**LDL SMALL**	**0.66**	GFR MDRD AFRICAN AM	0.37	WHITE CELL COUNT	0.41	LDL SMALL	0.32
**HOMA IR**	**0.66**	PLATELET COUNT THOUSAND	0.31	DHA	0.40	TRIGLYCERIDES	0.29
**WHITE CELL COUNT**	**0.56**	LDL SIZE	0.23	GLYCOHEMOGLOBIN A1C	0.33	BASOPHILS	0.23
**TOTAL NEUTROPHILS AB**	**0.52**	CRP HIGH SENSITIVITY	0.20	CRP HIGH SENSITIVITY	0.32	LYMPHOCYTES ABSOLUTE	0.20
URIC ACID	0.50	GLOBULIN	0.18	HOMA IR	0.30	EPA	0.19
LDL PARTICLE NUMBER	0.47	TOTAL NEUTROPHILS AB	0.16	RDW	0.30	Triglyceride HDL Ratio	0.19
DPA	−0.31	URIC ACID	−0.40	RED CELL COUNT	−0.29	DPA	−0.19
HDL PARTICLE NUMBER	−0.32	RED CELL COUNT	−0.42	A G RATIO	−0.30	RED CELL COUNT	−0.23
MCV	−0.33	CREATININE ENZ SER	−0.42	LINOLEIC ACID	−0.31	MONOCYTES ABSOLUTE	−0.24
VITAMIN D 25 OH TOT	−0.37	**HEMATOCRIT**	−**0.57**	LDL CHOL CALCULATION	−0.33	WHITE CELL COUNT	−0.25
EPA	−0.42	**HEMOGLOBIN**	−**0.61**	MCHC	−0.36	HEMATOCRIT	−0.26
DHA	−0.48	**DHA**	−**0.61**	HEMATOCRIT	−0.41	HEMOGLOBIN	−0.28
OMEGA 3 INDEX	−0.48	**EPA**	−**0.62**	ALBUMIN	−0.41	BILIRUBIN TOTAL	−0.28
OMEGA 3 TOTAL	−0.48	**DPA**	−**0.66**	OMEGA 6 TOTAL	−0.43	ARACHIDONIC ACID	−0.37
LDL SIZE	−0.50	**OMEGA 3 TOTAL**	−**0.69**	OMEGA 6 OMEGA 3 RATIO	−0.44	TOTAL NEUTROPHILS AB	−0.40
**HDL CHOL DIREC**	−**0.63**	**OMEGA 3 INDEX**	−**0.69**	HEMOGLOBIN	−0.47	**TOTAL NEUTROPHILS**	−**0.51**

The table contains the top 10 positively correlated analytes (upper part of the table) and the top 10 negatively correlated analytes (bottom part) for every archetype. Presented are the analyte names next to the correlation coefficient (r). Highlighted |r| >=0.5.

The second PC is mostly correlated with omega-3 fatty acids, including total omega-3’s, the individual omega-3’s DHA and DPA, and the omega-6/omega-3 ratio. In a typical western diet, the richest source of omega-3 fatty acids often comes from supplement use. Additionally, in our cohort supplement uptake is more prevalent in the older portion of the population, and therefore these findings suggest that the second axis is determined by supplement uptake or age. Since both age and supplement uptake are associated with this PC and between themselves, we cannot infer causality or determine whether one attribute is dominant over the other. The third and the fourth^th^ PCs are mainly correlated with cholesterol. As higher values of cholesterol are more frequent in men^52,53^, it suggests that the 3^rd^ split of data is based on gender. The first four PCs explain 33.65% of the variance in the data, and higher PCs were less conclusive. This analysis supports the previous characterization of the archetypes using the enrichment analysis, and provides hierarchy to the rules that shape the data. The data first splits according to wellness state, then supplement uptake/age, and lastly—by gender (Fig. 5). Interestingly, sex differences diminish with age and sickness.

### Utilizing longitudinal data and the movement on the tetrahedron for early detection of transitions from health to disease state

After finding the tetrahedron and characterizing the archetypes and the tradeoffs in the system, we used longitudinal data to study how individuals move on the tetrahedron over time. Moving towards the unhealthy-archetype is associated with higher levels of diabetes, obesity, and cardiovascular disease markers including insulin, glucose, LPIR, triglycerides, LDL. Therefore, advancing towards this archetype indicates possible deteriorating health. In contrast—moving away from the unhealthy-archetype towards the healthy plane suggests improvement in wellness. Indeed, when we correlated the distances between the archetypes and health markers such as weight and BMI, we obtained positive correlation with the older-archetype, and anti-correlation with the unhealthy-archetype (Supplementary Fig. 8).

The current dataset does not include electronic health records. Clinical information was obtained by self-reports, which included 113 adverse events of which 80 were unique events. The most common event was “kidney stones” that was reported by five participants (0.16% of the cohort, Supplementary Dataset 4), and therefore there are not many replicate trajectories for any specific disease in the current study as there are for common drug usage (e.g., statins) or for out-of-range values on risk biomarkers (e.g., LDL cholesterol, HbA1c). However, comparing the longitudinal measurements of individuals to their initial position allows detecting consistent change over time in a personalized (N of 1) manner”.

**Fig. 6:**
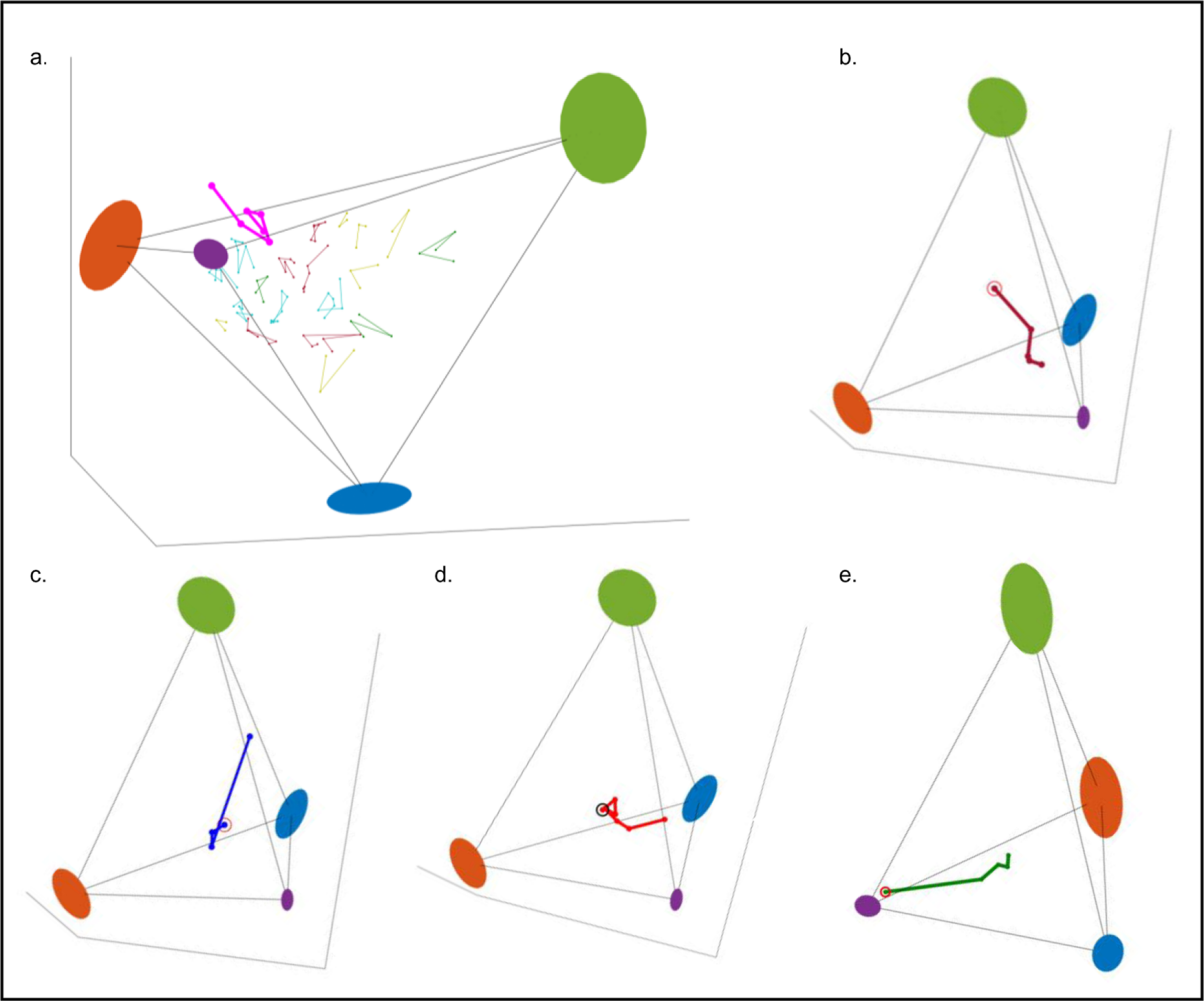
Trajectories of individuals and the movement on the tetrahedron can be used for early detection of transitions from health to disease state. **a** most individuals tumble around their initial position on the tetrahedron. Shown in the Figure are a few examples of trajectories, colored according to the number of timepoints: yellow- 3, green-4, red-5 and 6, and turquoise- 7 timepoints, the initial position is marked with a black circle. The pink trajectory belongs to a 64-year-old woman who was diagnosed with stage 3 bladder cancer prior to her blood measurements, her trajectory exceeds the boundaries of the tetrahedron. **b** Most of the trajectories are moving away from the unhealthy- archetype (green) towards the older and the healthy archetype (purple), as expected from a wellness program. Shown in the Figure an example of such a trajectory. **c** The trajectory of a 56-year-old woman that significantly progressed in her 4^th^ time-point (Euclidean distance: 13.2) towards the unhealthy-archetype (green) and closer to the male-archetype (blue), 3 days prior to a diagnosis of enlarged liver, gallbladder and pancreas. **d** The 7 timepoints trajectory of a 61-year-old woman, tumbling in the middle of the tetrahedra in the first 3 timepoints, and starting from the 4th time-point, gradually moving horizontally away from the female-archetype (orange) towards a point between archetype 1 and 3. Between her 6^th^ and 7^th^ visits she was diagnosed with Gallstones and Fatty liver disease. **e** A gradual trajectory of a 56-year-old man, who was not diagnosed with any disease, but consistently moves away from the healthy and the older archetype (purple) towards the center of the tetrahedron, and closer to the unhealthy archetype.

There are 1186 individual trajectories of three or more time-points (Supplementary Fig. 13), and participants move in all directions. The maximal Euclidian distance between two time-points is 19.4 (min: 0.1, mean: 2.5, Supplementary Fig. 9). Most participants tumble around their initial position, such that the mean Euclidean distance between the first and last visit is 3, (min: 0.3, max: 13.5, median: 2.7 std: 1.7, Supplementary Fig. 10, Fig. 6), however some participants significantly changed their position on the tetrahedron. Out of the top 2% of participants that significantly changed their position, 87% (20/23) were getting closer to the healthy and the older-archetype, and were moving away from the unhealthy-archetype as expected from a wellness program.

Three participants demonstrated a different pattern: a 56 years old woman who moved on her 4^th^ visit from the center of the tetrahedron towards the unhealthy-archetype (Euclidean distance: 13.2, Fig. 6). Three days after her 4^th^ blood draw she was diagnosed with enlarged liver, gallbladder and pancreas. Interestingly, she also moved closer to the male-archetype, showing that in abnormal situations a female can move toward the male-archetype, and that this unexpected movement might indicate an abnormal health status. This trajectory was ranked second in the length of the movement.

A similar case was detected for a woman in her 60 s that had seven timepoints, and was ranked 6th in trajectory length. In the first three time points she is tumbling in the middle of the tetrahedron and from the 4^th^ time-point onwards she is moving horizontally away from the female-archetype toward the point between the male and the older archetypes, with no vertical movement towards the unhealthy- archetype (Fig. 6). Between her 6^th^ and 7^th^ visits she was diagnosed with gallstones and fatty liver disease. Unlike the first example, the movement on the tetrahedron was gradual.

The second movement towards the unhealthy-archetype was observed for a 56 years old man whose initial position is very close to the older-archetype, and in all of his four following timepoints he consistently moved away from this archetype and toward the center of the tetrahedron (total change in Euclidean distance = 12.8). The participant’s measurements fall in the middle of the population distributions, and there is no record of diagnosis of any pathology, however many analytes are gradually changing in a consistent way, including insulin (from 3.9 to 6.2) and LDL small (from 90 to 143), which might indicate an evolving underlying condition. This trajectory was ranked 3rd in length.

The third case of such a movement, ranked 1st in length and belongs to a 54-year-old man, where three of his five timepoints crossed the tetrahedron boundaries, however there was no report of adverse events for this participant. Crossing the tetrahedron boundary and moving away from it means that the individual has an atypical set of values that are very different from the background distribution that was used to construct the tetrahedron, which can be due to error in the measurement, or may indicate an abnormal physiological condition.

Overall, 30 trajectories (2.5%) had a time-point that exceeded the tetrahedron boundaries, but there was only one trajectory that had 3 time-points that were out of the convex hull (described above) and another one that had 5 of 6 timepoints outside the simplex. This trajectory belongs to a 64 years old woman who was diagnosed with stage III bladder cancer, prior to these measurements (Fig. 6). Most cancers are undetectable through typical clinical lab tests. The measurement of specific proteins is usually used as biomarkers for different types of cancers^54–58^. Interestingly, all clinical labs measurements for this participant fall in the center of the distribution for all analytes in all visits (Supplementary Fig. 12). However, using longitudinal data and drawing her personal trajectory on the tetrahedron revealed unusual movement, which might indicate an underlying condition. Despite the limitation of the dataset, these few examples demonstrate how personal trajectories and the movement on the tetrahedron can be used to detect transitions from health to disease states, and vice versa, even when other computational and statistical tools show no indication of such a transition.

## Discussion

In this study we applied ParTI to high-dimensional human wellness data to aid in analyzing and visualizing the most dominant tradeoffs that shape the clinical labs data. The key findings are as follows: (1) ParTI analysis revealed that the clinical labs data fall on a statistically significant tetrahedron, with four archetypes. (2) Enrichment analysis of associated multi-omics and lifestyle data revealed characteristics of each of the archetypes: (i) the older and healthy archetype, (ii) the young and healthy females, (iii) the young and healthy males (iv) the unhealthy-archetype. (3) We then describe the clinical lab profiles at the archetypes, and found the major axes of variation and their hierarchy: (i) the wellness, (ii) the age, and (iii) the gender axis. (4) We found that the male-archetype shares more enriched features with the unhealthy-archetype than did the female or the older archetypes, which appeared to be due to generally less healthy lifestyle and dietary habits. (5) We found that sex differences diminished with age and in an aberrant health state. (6) Lastly, we characterized the movement of individual participants on the tetrahedron over time and found that most participants tumble around their initial position, and that the vast majority of the participants that showed a significant change in their trajectory, moved towards the healthy and older archetype, and away from the unhealthy archetype, as might be expected from a wellness program. (7) We then detected all the cases of participants that showed a different type of movement, and demonstrated how the movement on the tetrahedron might be utilized for monitoring individual participants and detecting signs of health transitions. Taken together, these findings demonstrated the power of geometry and dimensionality reduction in analyzing and visualizing high-dimensional datasets in a continuous trait space, and their capacity to be leveraged for monitoring individual’s health through blood measurements.

The four identified archetypes were strongly reflective of major aspects of physiology: females, males, older and unhealthy, as well as the tradeoffs and the major axes of variation. Interestingly, there were three wellness states and only one aberrant health state. In a different cohort (e.g., the clinical labs of breast cancer participants) there might be a different set of archetypes, axes of variation and different hierarchy of the tradeoffs, though the axes of variation that come out of this analysis are generally well known to have significant effects (sex, age, general health).

Moreover, one might ask why the clinical labs matrix was used to construct the simplex and not the metabolomics or proteomics data. We chose the clinical labs because it was the data type for which we had the largest number of observations, with standardized and commonly used measurements that have known interpretations. We also found that the simplex signal was the strongest for the clinical labs. Additionally, the clinical labs are the only dataset that is currently being measured in the clinic, and therefore the most relevant and applicable for the longitudinal analysis of an individual’s health trajectory in the resulting tetrahedron.

The enrichment analysis using different data types allowed us to characterize the four archetypes across multiple aspects, which were generally concordant and provided a unified view. For example, the unhealthy-archetype was enriched for high BMI, its corresponding PRS, high levels of the protein leptin, and various other traits like high blood pressure, low levels of physical activity, and poor dietary habits. Similar cohesion was seen for the other archetypes as well. Such traits represent sets that make sense and are fairly well understood to be interconnected, but others emerge that are not as known, such as the depletion and the enrichment of specific gut microbial genera associated with each archetype. Additionally, we provided an overview of the most apparent tradeoffs between archetypes that were significant after correcting for multiple hypothesis testing. The full enrichment tables contain distinct signatures for every archetype (Supplementary Dataset 2).

We analyzed the individual trajectories of participants, and demonstrated how it can be used for detecting unexpected motions. There are various ways to analyze the trajectories and define what is a significant movement, while excluding outliers and errors in measurements. For that purpose, we considered only trajectories that had at least three timepoints, and calculated the Euclidean distance between the initial and the end position. This analysis revealed three trajectories that had a significant change, and moved closer to the unhealthy archetype, and were described in detail in the Results. The second criterion for “abnormal” trajectory was the number of timepoints in a trajectory that were outside the tetrahedron boundaries. This analysis revealed only two trajectories that had more than one time point outside the tetrahedron (a single time point may indicate an error in the measurement or a transient state). Three out of the total five examples that had a unique trajectory according to these two criteria, self-reported an adverse event. Interestingly, the two women that moved away from the female-archetype, and closer to the male-archetype, both reported an adverse event that included the liver and the gallbladder, which fit the notion that young healthy males have a distinctive signature from females of lipids and markers of kidney function. Since the Arivale dataset does not contain the participant’s clinical records, valuable information for this kind of analysis might be missing. This might explain the two unique trajectories that do not have information about a specific diagnosed clinical condition. These two trajectories belong to male participants and the male-archetype was also enriched for missing information in the self-reported questionnaires. However, this analysis revealed the distinct trajectory of the participant that was subsequently diagnosed with bladder cancer. This example is noteworthy because it is the only example of a trajectory where 5/6 timepoints exceeded the boundaries of the tetrahedron. This is even more exceptional considering that this participant’s blood measurements individually fell within the distribution of the cohort, such that in outlier analysis, none of the individual measurements would have been abnormal. Moreover, usually cancers (other than leukemia) are not detected in standard clinical lab measurements. In the Arivale cohort there were several other cases of participants that were diagnosed with cancer or other diseases, but the transition was not captured by this analysis. This might be because of missing data, because the transition occurred before the participant joined the program, because the transition is not reflected in the clinical labs, or because of lack of sensitivity in the method. To determine to what extent this analysis could be further developed for detecting transitions, for calculating its sensitivity and specificity, as well as for correlating the archetypes with long-term health outcomes, a larger and more longitudinal dataset would be needed.

Taken together, this study implements a high order data representation of multi-omics measurements. Despite its limitations, it provides insights into the interplay between wellness and disease in deeply phenotyped data clouds. This work can help in characterizing disease transitions, and their reflection in the blood, and perhaps suggest a unique way to interpret blood tests.

## Methods

### Data collection

The de-identified data for consenting individuals was collected by Arivale incorporation as part of a scientific wellness program between 2015–2019. Participants in the program gave blood and stool samples and based on the measurements and their personal goals were guided by professional coaches how to change their lifestyle (dietary, exercise, sleep, supplement taking and stress management) in order to improve their health. There were 3,558 participants in the program, and samples were collected approximately every 6 months. The number of time-points per participant vary between 1 to 8 time points. The distribution of the time points and the demographic description of the cohort is described in the SI. The study was approved by the Western Institutional Review Board (WIRB) with Institutional Review Board (IRB) study number 20170658 at the Institute for Systems Biology.

### Clinical laboratory tests

Blood draws for all assays (metabolomics, proteomics and clinical labs) were performed at LabCorp service centers. At every blood draw, weight and height were measured and BMI was calculated using the formula: (weight(kg))/(height(m))^2^. Participants were requested to avoid alcohol, vigorous exercise, aspartame and monosodium glutamate 24 h prior the blood draw, and fast 12 h in advance. Participants were asked to declare if they were fasting as directed, and negative answers were used as exclusion criteria. Another exclusion criterion was based on ethnicity. Different ethnicities have different ranges of clinical labs, however, there was no good representation of ethnicities other than white (82%), and to avoid the natural grouping by race (which happened prior to the inclusion criteria with the 2% of Asians that were clustered next to a specific archetype), only participants that declared one of the following: white, Ashkenazi Jewish, Sephardic Jewish, Hispanic Latino or Spanish origin were included in this study. No further genetic validation was done to confirm these statements. Excluded also participants and analytes that had more than 10% missing values, resulting in a dataset of 3094 individuals and 67 analytes.

### Data selection and normalization

To avoid skewed results due to data multiplication (multiple visits per participant), one visit was randomly selected for each individual using the “randi” function in MATLAB. The data selection process was repeated 7 times, and every time the randomly selected data was used to find a tetrahedron. In 5 out of 7 repetitions a significant tetrahedron was found (*P*-value < 0.05), as shown in Fig. 2. The significance test was done as described in Hart et al.^25^. One of the data selections that had a *P*-value < 0.001 was then used for all further analysis, such that for every participant there was a key of participant internal ID and visit, and that key was used to match samples in all other datasets—proteomics, metabolomics etc. Missing values were imputed by the analyte mean and the clinical labs dataset was Z-normalized following the data selection and prior to subsequent data analysis steps.

### Polygenic risk scores (PRS)

52 polygenic risk scores (PRS) were calculated as a continuous measure of risk aggregating the effects of multiple SNPs, as described in Zubair et al.^11^. Briefly, each of these polygenic scores was constructed using publicly available summary statistics from published Genome-Wide Association Studies (GWAS)^59–61^. After FDR correction and filtering correlated SNPs, the PRS for each individual was calculated by summing up the published effect size for each selected SNP multiplied by the number of effect alleles the individual carried for that SNP, across all of the selected SNPs. The PRS were used for enrichment analysis and no imputation for missing values was carried out.

### Proteomics

Plasma protein levels were measured by Olink Biosciences in 3 panels: Cardiovascular II, Cardiovascular III and Inflammation, the data was processed and batch corrected as described in Wilmanski et al. 2019^13^. The proteomics dataset was matched to the clinical lab dataset and included the same 3094 participants and visits, and 265 proteins that were measured from the same blood draws as the clinical labs. The proteins dataset was used for enrichment analysis and no imputation for missing values was carried out.

### Metabolomics

Metabolites from plasma samples were assayed by Metabolon (North Carolina). Untargeted metabolomics analysis was performed on plasma extracted from whole blood using Metabolon’s ultra-high-performance liquid chromatography/tandem mass spectrometry (UHPLC/MS/MS) Global Platform (Ryals et al. 2007). Sample handling, quality control, and data extraction, along with biochemical identification, data curation, quantification, and data normalizations have been previously described^13^. A total of 990 different plasma metabolites were measured for each individual and matched to the same blood draws as in the clinical labs dataset. This dataset was used in the enrichment analysis and no imputation was carried out.

### Microbiome

Stool specimens were taken at participants’ homes using a standardized kit supplied by Second Genome or DNAgenotek. The samples were processed and analyzed as described in Wilmanski et al., 2019^13^, and matched to the same blood draws that were used in other datasets. Three diversity measurements (Shannon, Chao1, Diversity of observed species) were calculated at the ASV level after rarefaction. For correlation of individual genera with archetypes, only genera that had less than 5% zero values and a mean greater than 5 counts were used (a total of 100 genera).

### Self-reported questionnaires and lifestyle information

Self-administered questionnaires were completed by the participants during their initial assessment. These questionnaires included the areas of: current health state, health history, dietary, exercise and activity habits, stress, mood and satisfaction surveys. Lifestyle habits were also recorded by Fitbit activity tracker which recorded the number of steps that the participants took every day, heart rate and sleep. These self-reported questionnaires and fitbit information were used for enrichment analysis and the characterization of the different archetypes.

### Fitting a tetrahedron to the clinical labs dataset using ParTI

To fit a tetrahedron to the multi-dimensional clinical labs dataset we used the ParTI software package in MATLAB^25^. The ParTI software fits a polyhedron to the data, finds the archetype position and calculates the significance of the fitted polyhedron and the error in the archetype positions. To determine the significance of the polyhedron, the software calculates the ratio between the polyhedron and convex hull of the data (t-ratio). Then the program shuffles the data and calculates the t-ratio for the shuffled data. ParTI repeats this process 1000 times and a *P*-value is calculated by counting how many times the t-ratio of the shuffled data was lower than the real data t-ratio, divided by the number of runs (1000). To choose the number of archetypes we ran the ParTI software with 2,3,4, and 5 vertices and chose the best *P*-value.

### Enrichment analysis

After finding the tetrahedron and the archetype positions, we used all other data types to characterize the archetypes by enrichment analysis as described in Hart et al. 2015^25^. In short, we were looking for traits that are high close to an archetype and as moving away from the archetype the trait decays. To calculate the enrichment of a trait close to an archetype we bin the data into 20 equal bins according to the distance from the archetype. For continuous variables like age, weight, heart rate, proteins, metabolites and others, we compare the mean and median of the trait in the first bin to their values in the rest of the data using *t*-test. For discrete variables, we calculate the hypergeometric probability. To determine if a *P*-value is statistically significant and avoid type 1 errors for multiple hypothesis testing we used the Bonferroni correction. We applied 12,848 tests and therefore we set the threshold for significance to be 0.05/12,848 = 3.8917e-06. We test every variable for every archetype, and we use all data points in every test. The full tables that summarize the enrichment analysis can be found in Supplementary Dataset 2.

### Enrichment analysis sensitivity to bin size

To determine the sensitivity of the enrichment analysis to the bin size we ran the analysis with 15, 20 and 25 bins. In general, when increasing the number of bins, fewer variables were found to be enriched with significant *P*-value after Bonferroni correction. With 15 bins, 53 (16%) additional features were significantly enriched compared to 20 bins, and 54 (16%) features did not pass the Bonferroni threshold when dividing the data into 25 bins. The full tables of features and their *P*-values can be found in Supplementary Dataset 3.

### Reporting summary

Further information on research design is available in the Nature Research Reporting Summary linked to this article.

## Supplementary information

Supplementary information

Reporting Summary

Dataset 1

Dataset 2

Dataset 3

Dataset 4

Dataset 5

Description of Additional Supplementary Files

## Data Availability

Data used in this paper is either (1) data owned by ISB (in affiliation with Providence St. Joseph Health), which will be made freely available for academic use; or (2) data generated by Arivale’s commercial service. ISB and Arivale have an Asset License Agreement, which gives ISB access to de-identified datasets from Arivale commercial subscribers. Per the agreement, ISB is not permitted to upload datasets from commercial subscribers to public databases. To facilitate collaborative validation and follow-up studies, ISB has created a Data Use Agreement (DUA) that governs use of the commercial datasets, and will make available any data used in publications to 3rd parties that contact ISB and agree to the DUA. The limitations are consistent with other DUAs in place by other controlled-access databases (e.g., dbGaP): that the recipient will not disclose the data to 3rd parties who themselves have not signed the DUA; the recipient will not attempt to re-identify the participants from their data; and that the recipient may only use the data for non-commercial purposes. Inquiries to access the data can be made at data-access@isbscience.org and will be responded to within 7 business days.

## Code availability

Code used for ParTI analysis, other analysis, and for generating the figures is available through the Hood-Price lab GitHub.
