# Supplementary material for: The geometry of clinical labs and wellness states from deeply phenotyped humans
Source: Nat Commun. 2021 Jun 11;12:3578. doi: 10.1038/s41467-021-23849-8 (PMC8196202; doi:10.1038/s41467-021-23849-8)
Supplement: Supplementary file 1 — Supplementary information [file 41467_2021_23849_MOESM1_ESM.pdf]

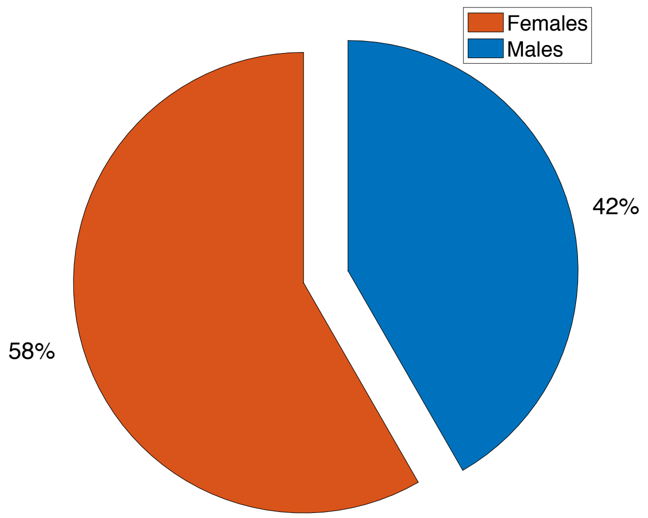

Supplementary Figure 1: Gender distribution: there are slightly more females (58%) in the cohort

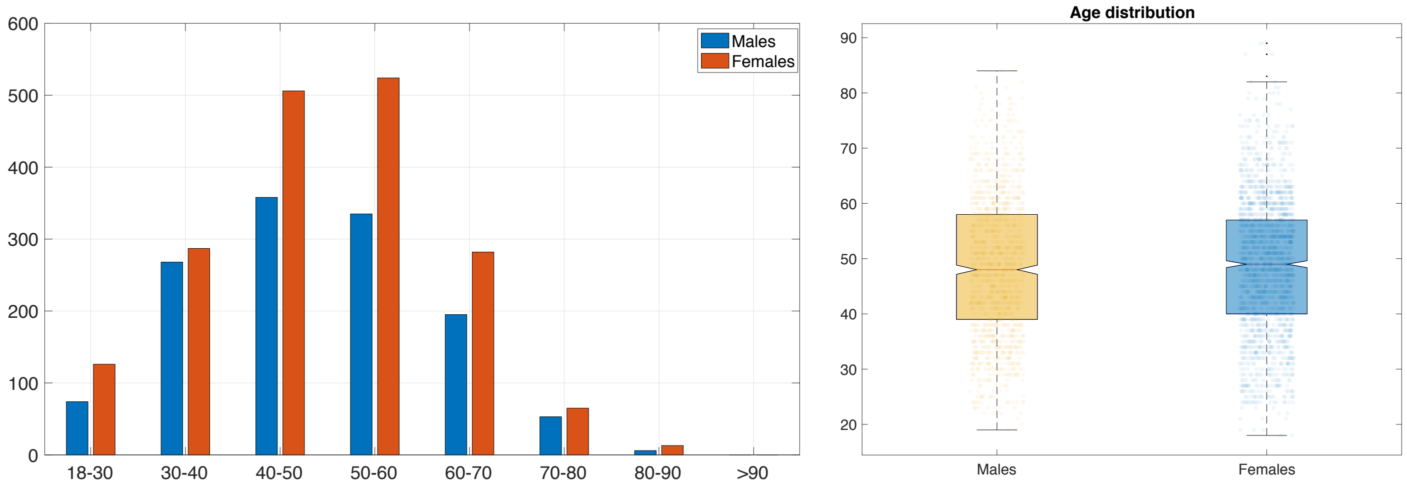

Supplementary Figure 2: Age distribution: the age is normally distributed for both males and females the black boxes on the right figure indicate the 25th and 75th percentiles, the colored dots are the cohort's age distribution, the black bars are the limits of the distributions (18-90), and the red line indicate the median.

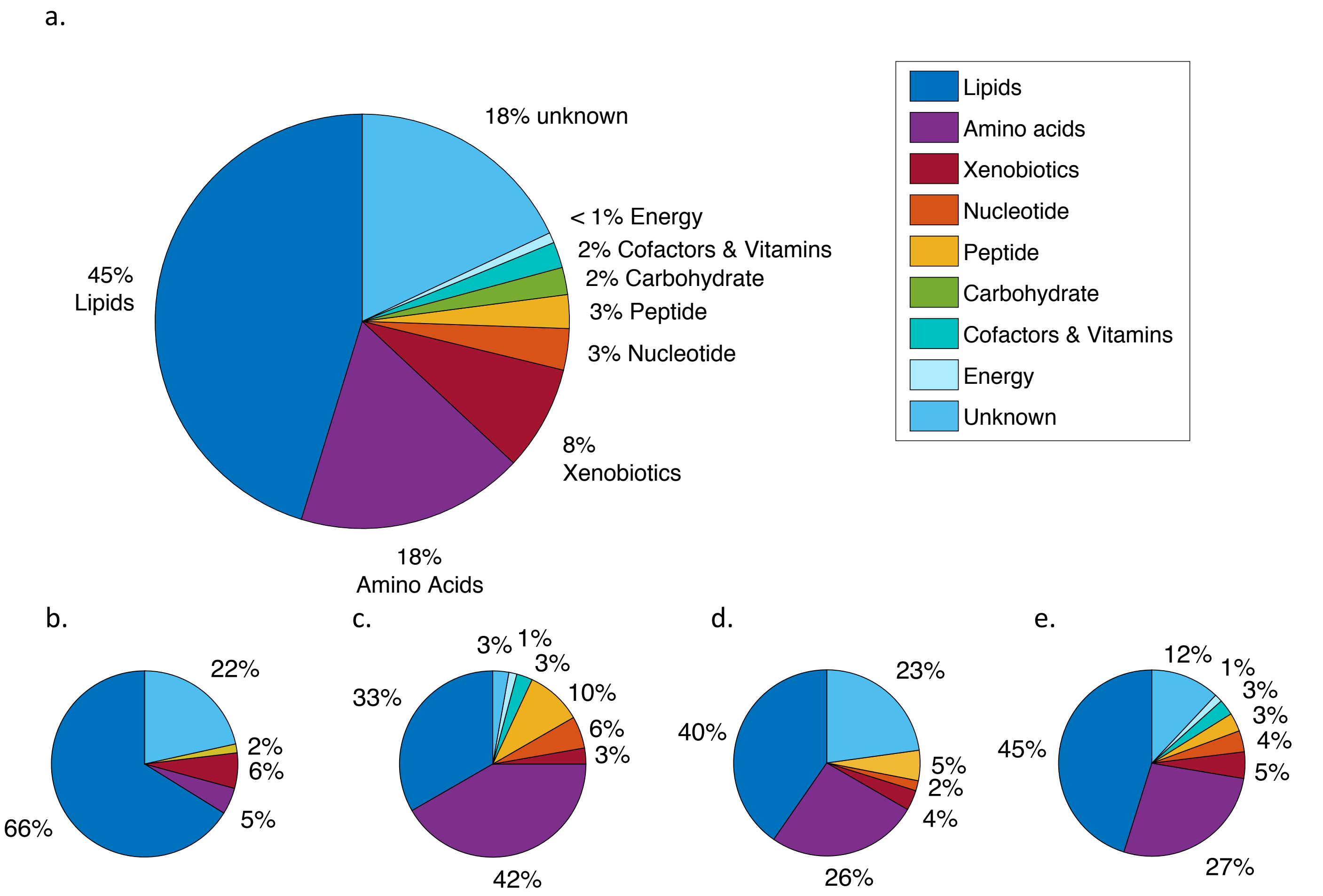

Supplementary Figure 3: a) The distribution of the 990 metabolites that were measured in the Arivale cohort. **b)** the distribution of the enriched metabolites for archetype 1, N=65. **c)** distribution of the enriched metabolites for archetype 2, N=72. **d)** distribution of the enriched metabolites for archetype 3, N=56. **e)** distribution of the enriched metabolites for archetype 4, N=216.

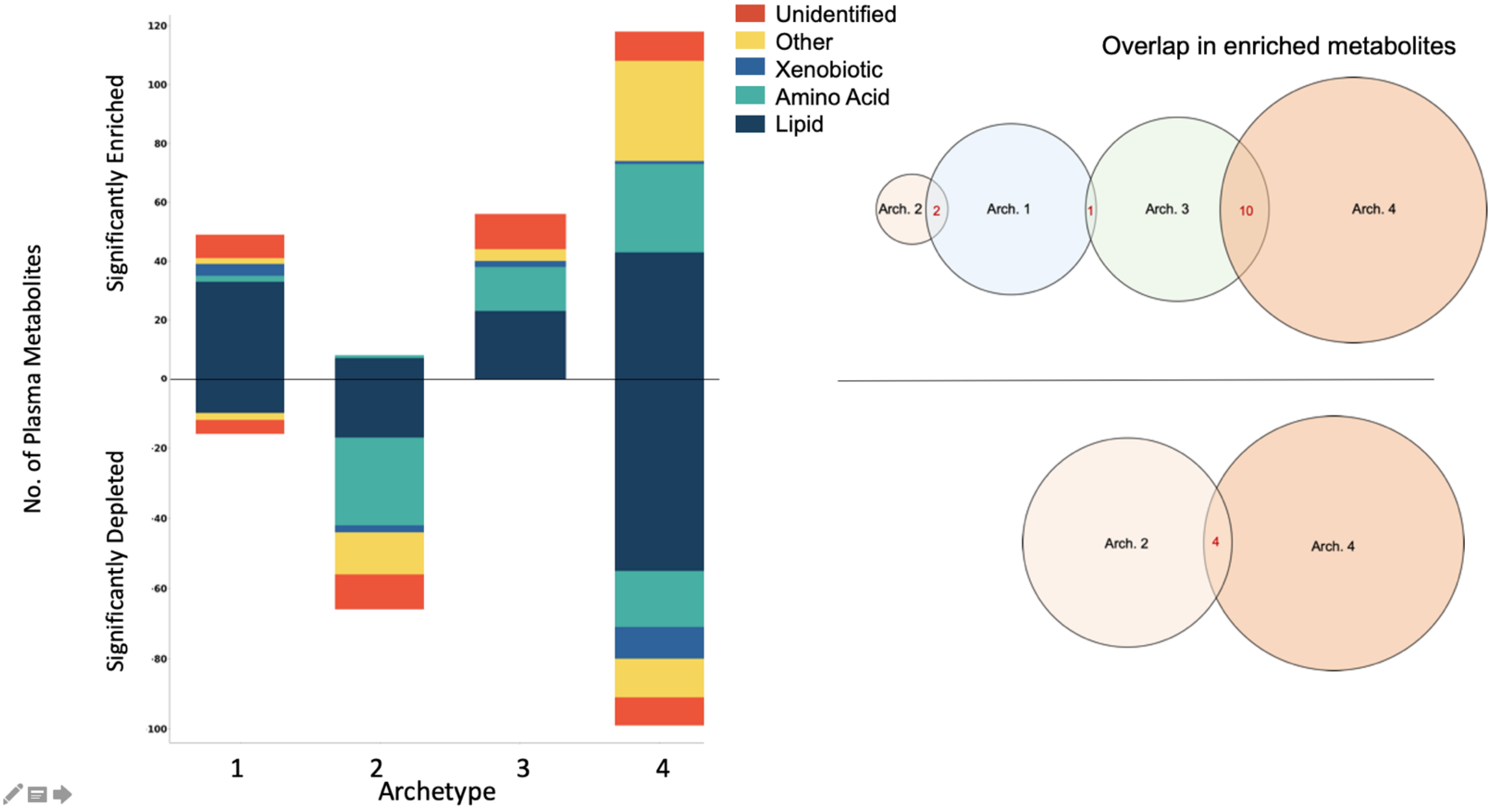

Supplementary Figure 4: There is a distinctive signature of enriched metabolites for every archetype, with very little overlap. Archetype number 4 is the most perturbed with 216 enriched metabolites, 98 of them found to be enriched with low levels.

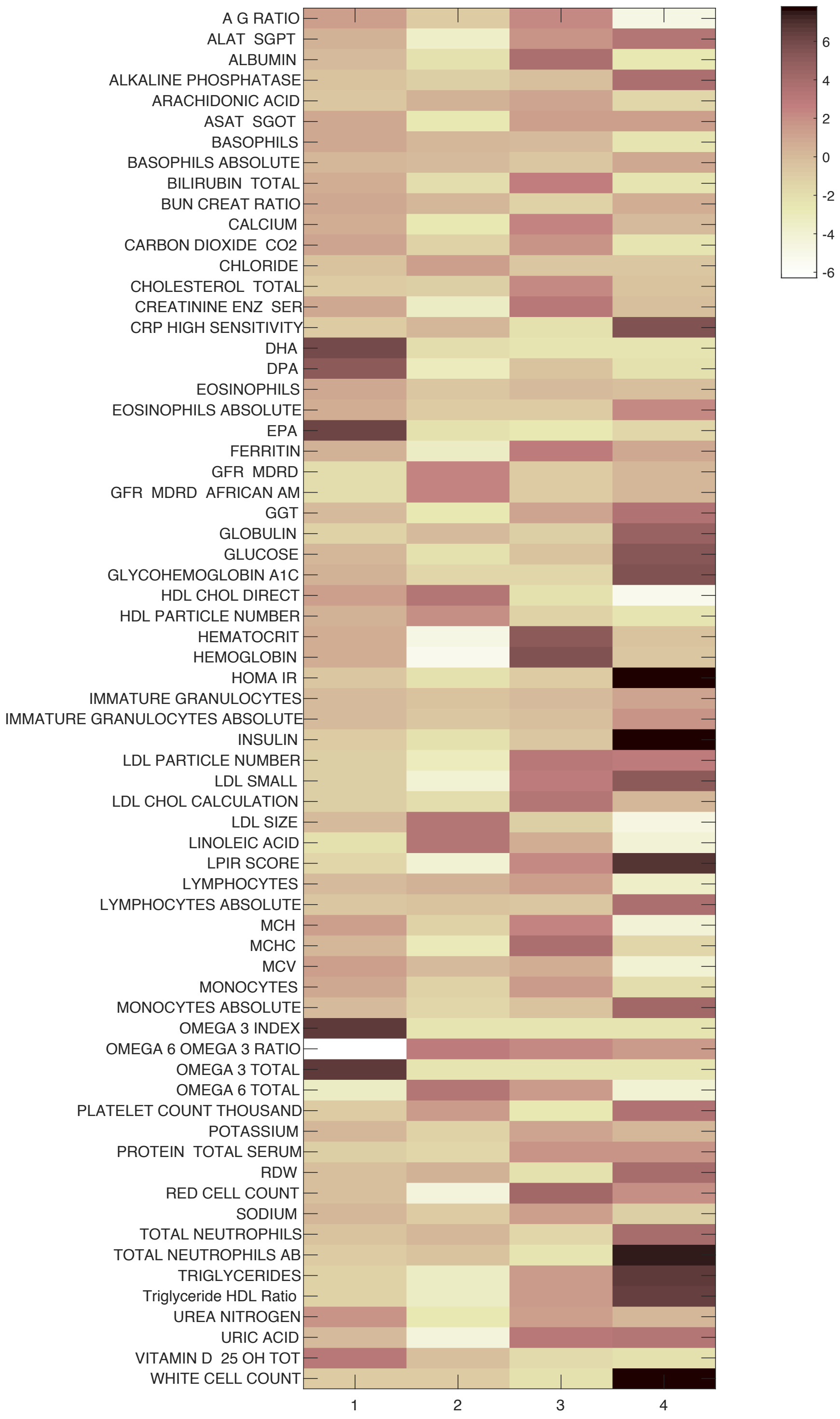

Supplementary Figure 5: The blood chemistries profiles at the archetypes

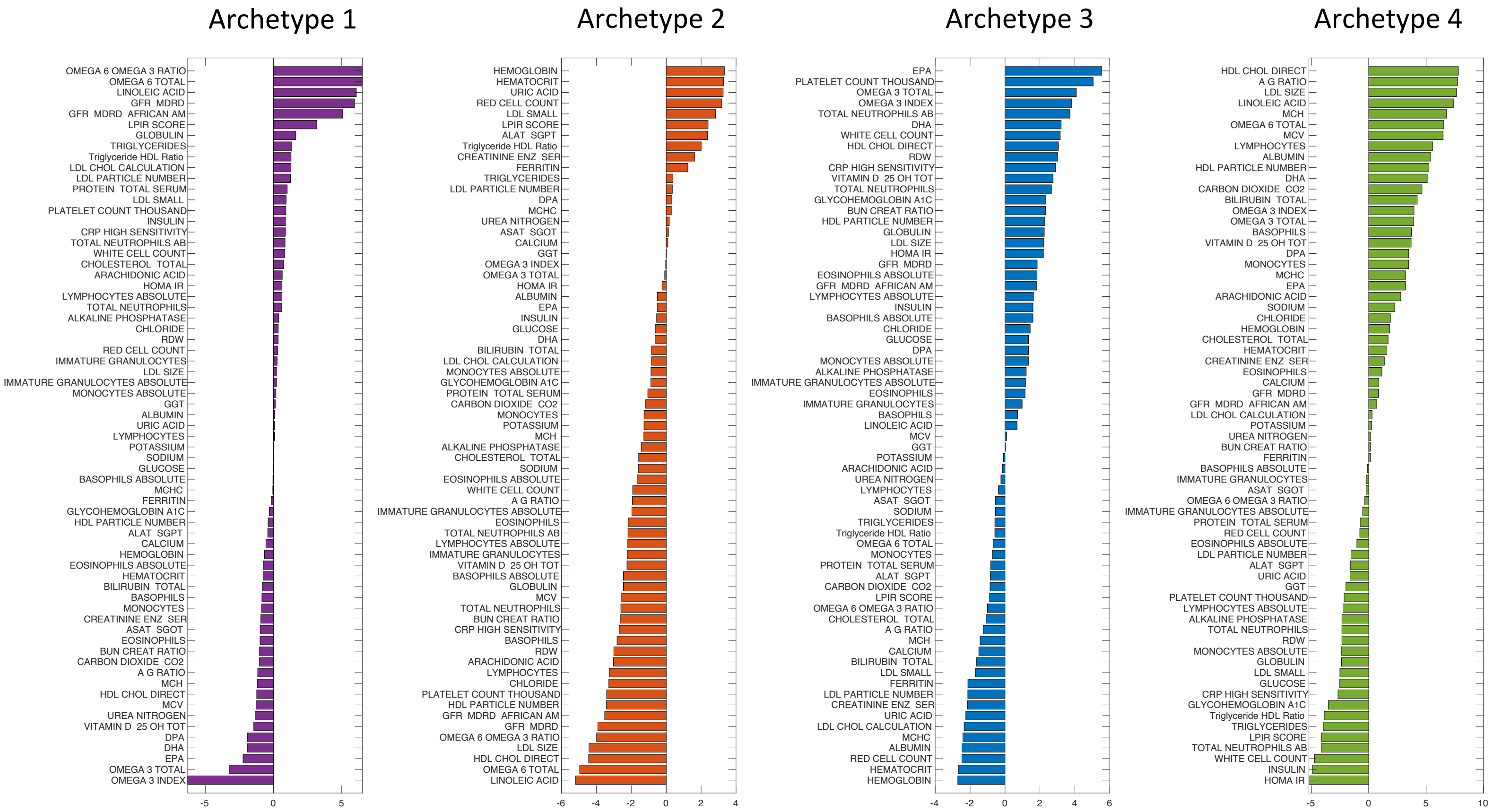

Supplementary Figure 6: Correlation between the distances from the archetypes and the analytes reveals the principal analytes next to every archetype. The distances between the data-points and the archetypes were correlated to the levels of every analyte. Positive correlation (R>0) means that low levels of the analyte correlate with shorter distances, anti-correlation (R<0) means that higher levels of the analyte correlate with shorter distances.

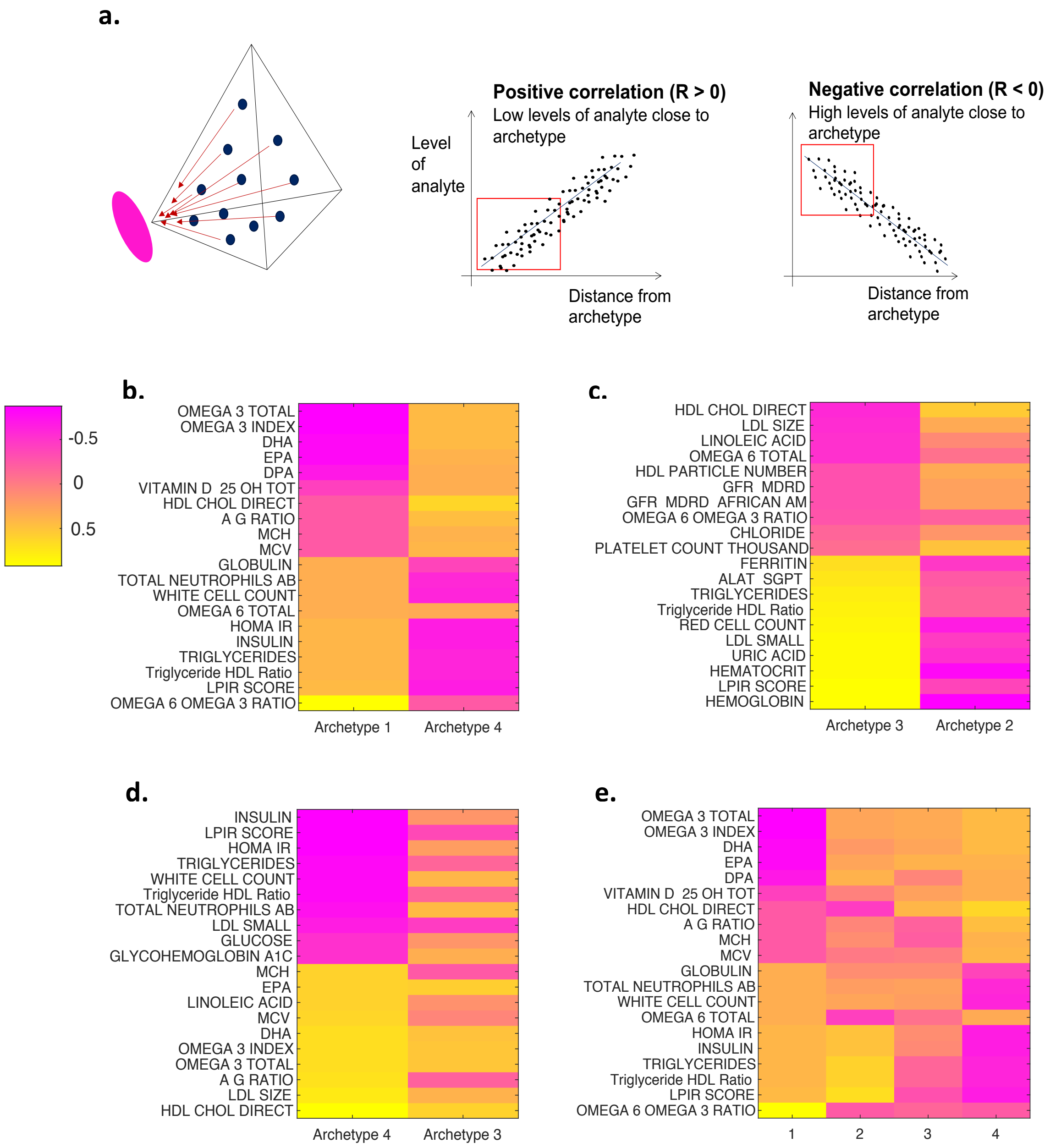

Supplementary Figure 7: Correlation between the distances from the archetypes and the analytes reveals the principal analytes next to every archetype, and the trade-offs in the data. a) schematic view: the distances between the data-points and the archetypes were correlated to the levels of every analyte. Positive correlation (R>0) means that low levels of the analyte correlate with shorter distances, anti-correlation (R<0) means that higher levels of the analyte correlate with shorter distances. **b)** the correlation coefficients for archetype 1 were ordered and the top 10 and bottom 10 correlations are presented (left column). The levels of Omega 3, DHA, EPA, DPA, Vitamin D are highly anti-correlated with distances from archetype 1, such that high levels are correlated with shorter distances (anti correlation at the upper rectangle). Omega 6/Omega 3 ratio, LPIR, Triglyceride/HDL ratio, Triglyceride and others are highly correlated with the distances such that low levels are closer to the archetype (lower rectangle). The right panel are the correlation coefficients for the same analytes with distances to archetype 4, demonstrating the clear trade-off between these two archetypes. **c)** the same as b) for archetype 2 and 3, analytes were ordered according to the correlation coefficients of archetype 3**. d)** the same as b) for archetype 4 and 3, analytes were ordered according to the correlation coefficients of archetype 4**. e)** comparison between the 4 archetypes, analytes were ordered according to archetype 1. The full table of the correlation coefficients can be found in Supplementary Table 2.

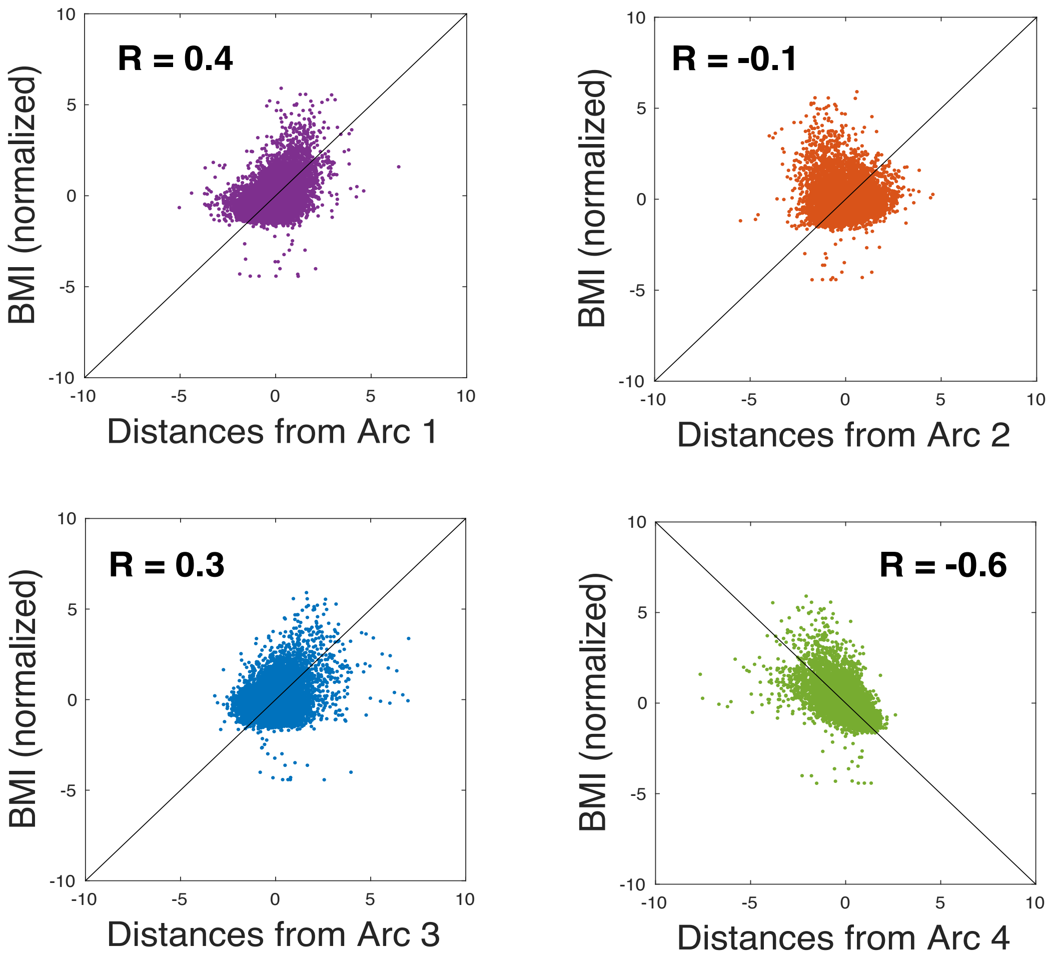

Supplementary Figure 8: the distances from the archetypes are correlated with health markers like BMI and weight. High BMI is correlated with shorter distances from archetype number 4 – the unhealthy archetype, low BMI is correlated with shorter distances to archetype number 1 – the healthy and older archetype.

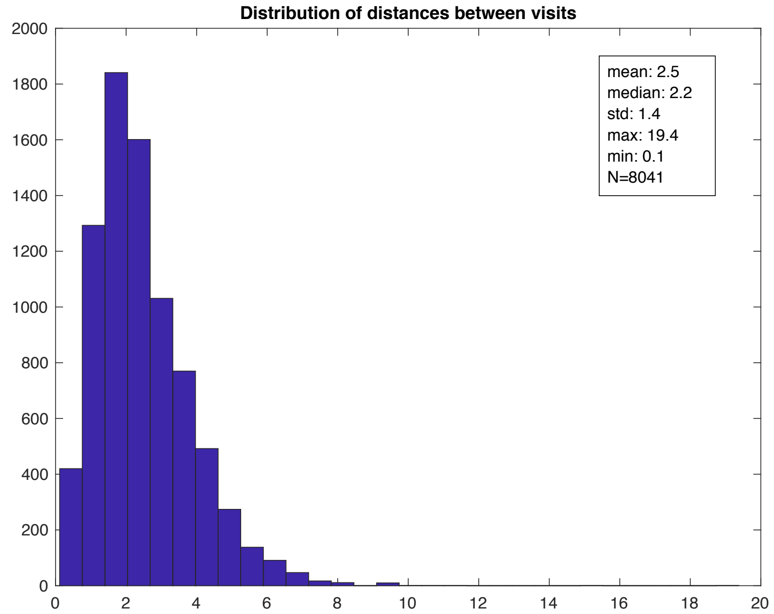

Supplementary Figure 9: The distribution of distances between two timepoints. Clients in this cohort had between 1 to 8 timepoints. For clients that had more than 1 visit we computed the position on the tetrahedron for every visit and then calculated the Euclidian distance between every two consecutive timepoints and drew the distribution.

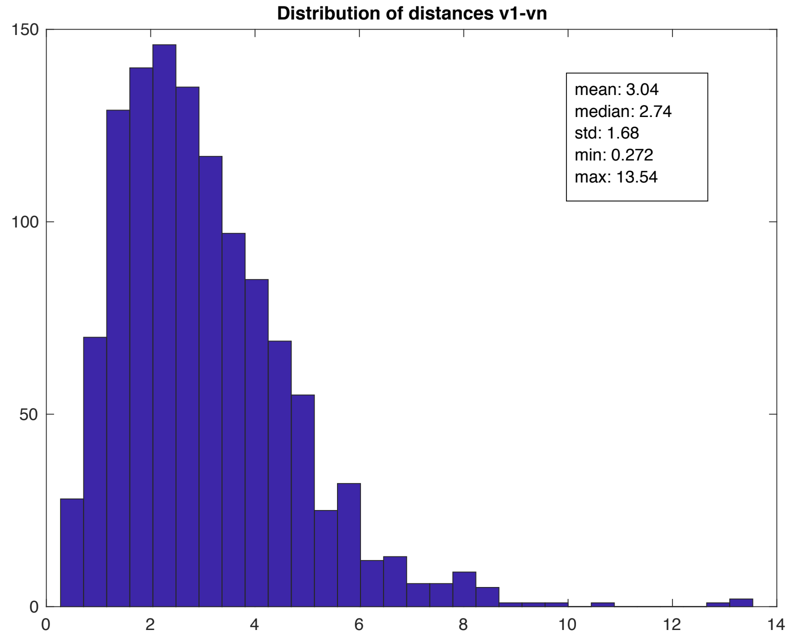

Supplementary Figure 10: The distribution of Euclidian distances between the first and last visit per client (N=1186).

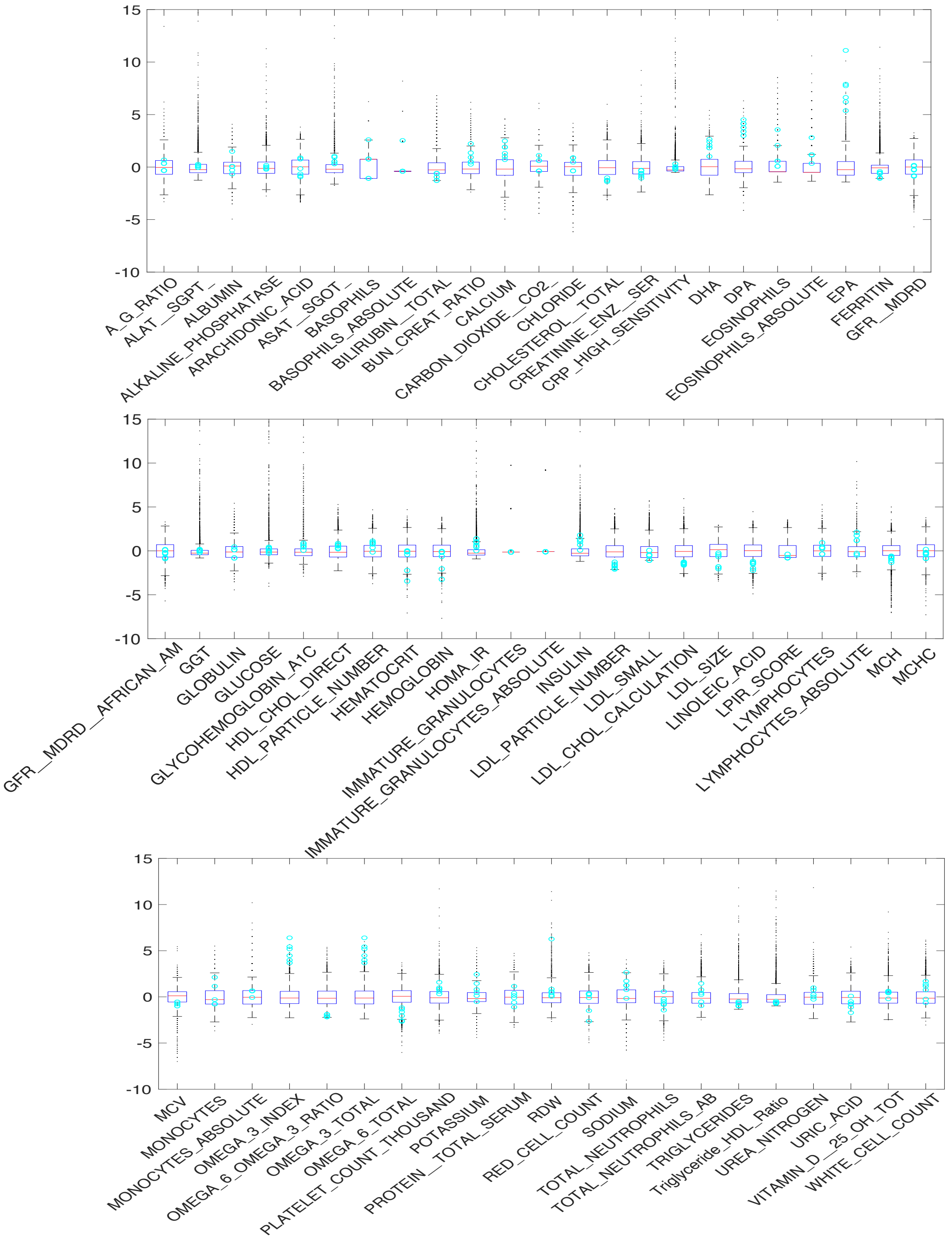

Supplementary Figure 11: The trajectory of the 64-years-old woman that was diagnosed with bladder cancer exceeds the tetrahedron boundaries even though most of her measurements fall in the middle of the distributions. Shown in the figures are the distributions of the 67 analytes, the blue boxes indicate the 25th and 75th percentiles, the black dots are the outliers, the black bars are the limits of the distributions, the red line indicate the median value, and the cyan circles indicate the values of the patient's blood draws in her 6 visits.

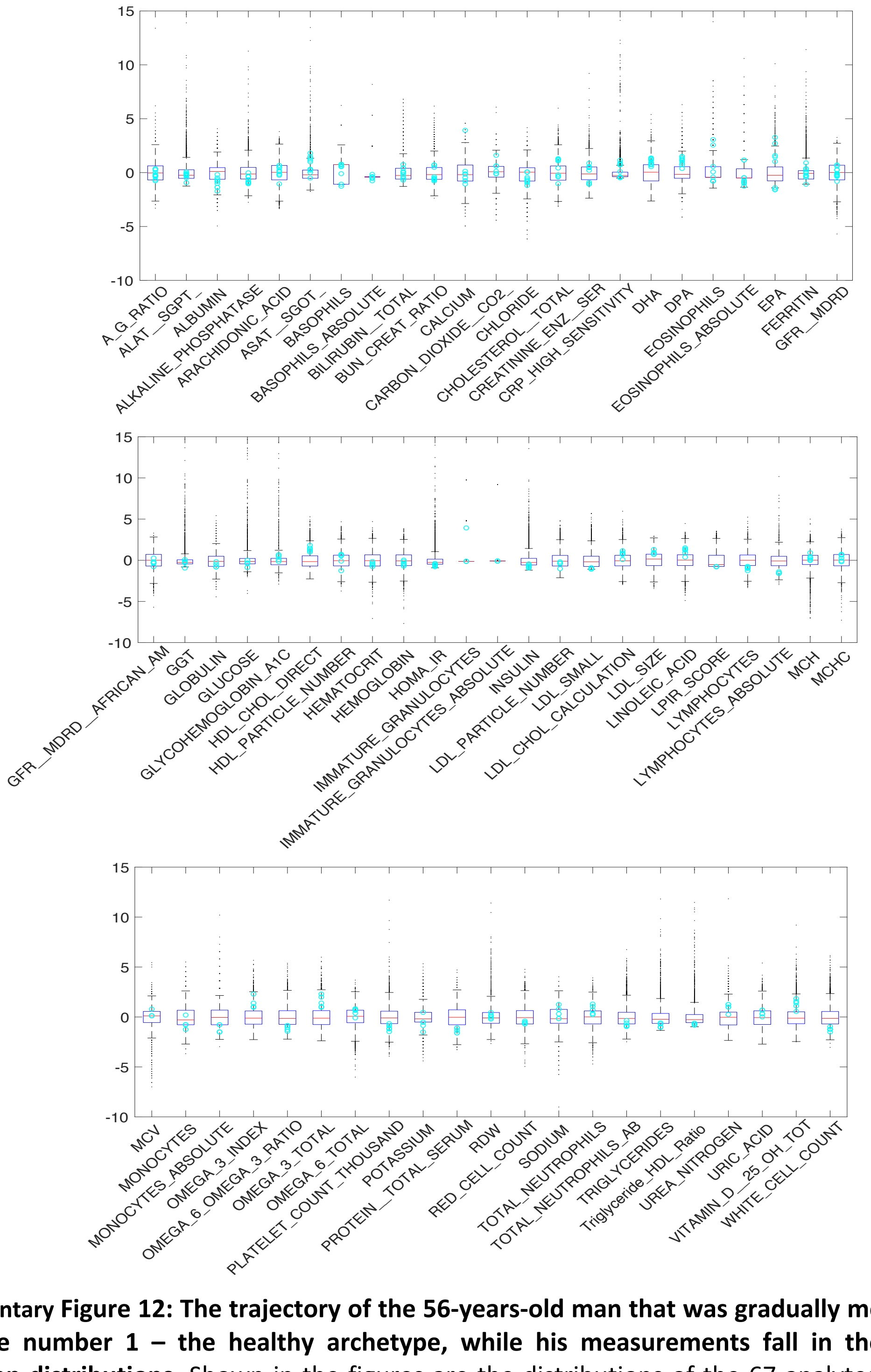

Supplementary Figure 12: The trajectory of the 56-years-old man that was gradually moving away from archetype number 1 – the healthy archetype, while his measurements fall in the middle of the population distributions. Shown in the figures are the distributions of the 67 analytes, the blue boxes indicate the 25th and 75th percentiles, the black dots are the outliers, the black bars are the limits of the distributions, the red line indicate the median value, and the cyan circles indicate the values of the patient's blood draws in his 5 visits.

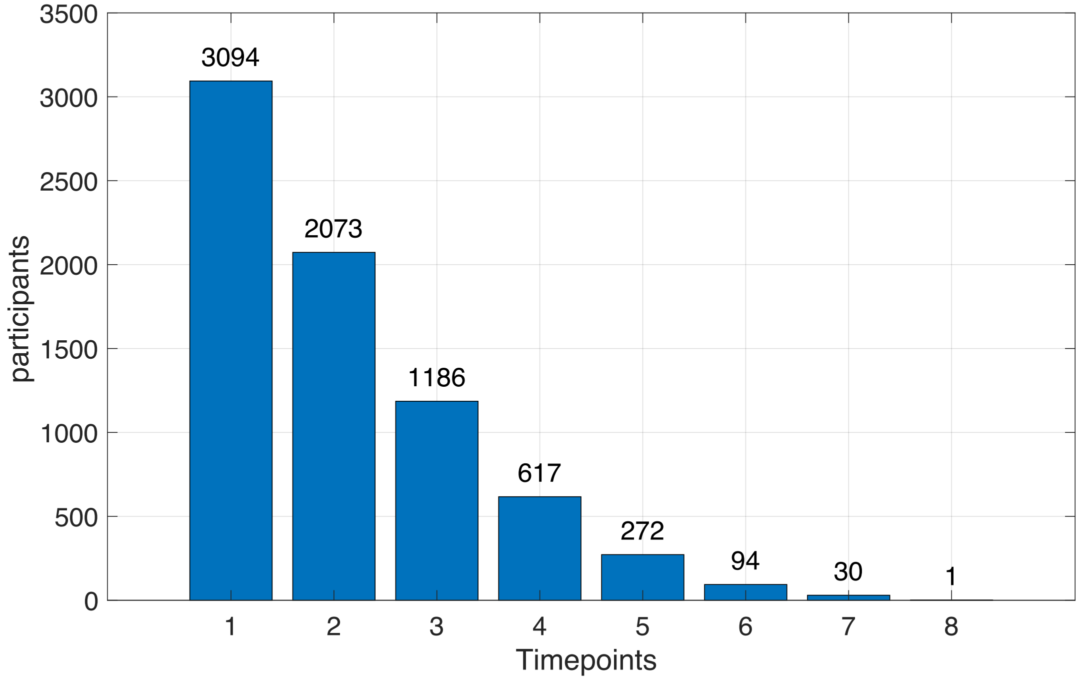

Supplementary Figure 13: The distribution of trajectory length. The 1186 trajectories that had 3 or more time-points were used for the trajectory analysis.
