# Supplementary material for: The geometry of clinical labs and wellness states from deeply phenotyped humans
Source: Nat Commun. 2021 Jun 11;12:3578. doi: 10.1038/s41467-021-23849-8 (PMC8196202; doi:10.1038/s41467-021-23849-8)
Supplement: Supplementary file 2 — Reporting Summary [file 41467_2021_23849_MOESM2_ESM.pdf]

nature research

Corresponding author(s): *Double-blind peer review submissions: write DBPR and your manuscript number here instead of author names.*

Last updated by author(s): *YYYY-MM-DD*

# Reporting Summary

Nature Research wishes to improve the reproducibility of the work that we publish. This form provides structure for consistency and transparency in reporting. For further information on Nature Research policies, see Authors & Referees and the Editorial Policy Checklist .

## Statistics

For all statistical analyses, confirm that the following items are present in the figure legend, table legend, main text, or Methods section.

| n/a | Confirmed | |
|---|---|---|
| ☐ | ☒ | The exact sample size ($n$) for each experimental group/condition, given as a discrete number and unit of measurement |
| ☐ | ☒ | A statement on whether measurements were taken from distinct samples or whether the same sample was measured repeatedly |
| ☐ | ☒ | The statistical test(s) used AND whether they are one- or two-sided<br>*Only common tests should be described solely by name; describe more complex techniques in the Methods section.* |
| ☐ | ☒ | A description of all covariates tested |
| ☐ | ☒ | A description of any assumptions or corrections, such as tests of normality and adjustment for multiple comparisons |
| ☐ | ☒ | A full description of the statistical parameters including central tendency (e.g. means) or other basic estimates (e.g. regression coefficient) AND variation (e.g. standard deviation) or associated estimates of uncertainty (e.g. confidence intervals) |
| ☐ | ☒ | For null hypothesis testing, the test statistic (e.g. $F$, $t$, $r$) with confidence intervals, effect sizes, degrees of freedom and $P$ value noted<br>*Give P values as exact values whenever suitable.* |
| ☒ | ☐ | For Bayesian analysis, information on the choice of priors and Markov chain Monte Carlo settings |
| ☒ | ☐ | For hierarchical and complex designs, identification of the appropriate level for tests and full reporting of outcomes |
| ☐ | ☒ | Estimates of effect sizes (e.g. Cohen's $d$, Pearson's $r$), indicating how they were calculated |

*Our web collection on statistics for biologists contains articles on many of the points above.*

## Software and code

Policy information about availability of computer code

| | |
|---|---|
| Data collection | Blood samples were collected by trained phlebotomists at LabCorp of America and Quest Diagnostics centers. Stool samples were collected by participants at their homes and processed by DNAGenotek or Second Genome. Gut Microbiome analysis was performed on FASTQ files using the publicly avialable QIIME pipeline 28 (version 1.9.1; default parameters) with closed-reference OTU picking against the Greengenes database (version 13_08). |
| Data analysis | Data was processed and analyzed using Python version 2.7/3.5+. Statistical analysis was performed using the Python scipy.stats and statsmodels packages. Machine learning algorithms were implemented using Scikit-learn packages. All code used to analyze the data and create the essential figures is deposited in a public GitHub repository: https://github.com/PriceLab/ShannonMets |

For manuscripts utilizing custom algorithms or software that are central to the research but not yet described in published literature, software must be made available to editors/reviewers. We strongly encourage code deposition in a community repository (e.g. GitHub). See the Nature Research guidelines for submitting code & software for further information.

## Data

Policy information about availability of data

All manuscripts must include a data availability statement. This statement should provide the following information, where applicable:

- Accession codes, unique identifiers, or web links for publicly available datasets
- A list of figures that have associated raw data
- A description of any restrictions on data availability

Data Availability
The model summary statistics for all metabolites, proteins and clinical labs analyzed are available to download in Supplementary Tables 2-4. Qualified researchers can access the full de-identified dataset for research purposes. Requests should be sent to: nathan.price@systemsbiology.org.

# Field-specific reporting

Please select the one below that is the best fit for your research. If you are not sure, read the appropriate sections before making your selection.

- [x] Life sciences
- [ ] Behavioural & social sciences
- [ ] Ecological, evolutionary & environmental sciences

For a reference copy of the document with all sections, see nature.com/documents/nr-reporting-summary-flat.pdf

# Life sciences study design

All studies must disclose on these points even when the disclosure is negative.

| | |
|---|---|
| Sample size | Sample size was not chosen for this study. Rather, inclusion criteria deemed necessary to investigate the proposed research questions were specified. The inclusion criteria were the main limiting factor in the number of participants used for this study. |
| Data exclusions | A total of 4 and 6 participants were excluded from the discovery and validation cohort, respectively. This is described clearly in the methods section of the manuscript. The rationale for removal of these participants was because they fell outside of 3 standard deviations from the mean. Like ordinary least square regression, penalized regression is sensitive to outliers which skews the generated models. That is why outliers were removed prior to analysis. |
| Replication | We attempted to replicate our findings by using a validation cohort of participants who enrolled into the Scientific Wellness program at an earlier time point and whose stool samples were analyzed by a different microbiome vendor. Many of our major findings were confirmed, while some (for example the moderate capacity of blood proteomics in predicting gut alpha diversity) were not. The manuscript describes the findings tested in the validation cohort and which of these were successfully replicated. |
| Randomization | Where appropriate, our statistical models were adjusted for covariates including sex, age, and BMI. The methods section of the manuscript describes the variables for which each regression model was adjusted. |
| Blinding | No randomization was performed, as well as no experimental intervention. Therefore, blinding was not performed in the analysis. |

# Reporting for specific materials, systems and methods

We require information from authors about some types of materials, experimental systems and methods used in many studies. Here, indicate whether each material, system or method listed is relevant to your study. If you are not sure if a list item applies to your research, read the appropriate section before selecting a response.

## Materials & experimental systems

| n/a | Involved in the study | |
|---|---|---|
| [x] | [ ] | Antibodies |
| [x] | [ ] | Eukaryotic cell lines |
| [x] | [ ] | Palaeontology |
| [x] | [ ] | Animals and other organisms |
| [ ] | [x] | Human research participants |
| [x] | [ ] | Clinical data |

## Methods

| n/a | Involved in the study | |
|---|---|---|
| [x] | [ ] | ChIP-seq |
| [x] | [ ] | Flow cytometry |
| [x] | [ ] | MRI-based neuroimaging |

## Human research participants

Policy information about studies involving human research participants

| | |
|---|---|
| Population characteristics | A detailed description of population characteristics is available in the submitted text in Table 1 and Supplementary Table 1. |
| Recruitment | Participants are self-enrolled in the Arivale program, since it is a subscription service. Researchers do not play a role in recruiting participants for the program. Upon entering the program, participants are provided with the option to permit the use of their de-identified data for scientific discovery. These participants then join the research cohort and are used for downstream analysis. |
| Ethics oversight | Procedures for the current study were run under the Western Institutional Review Board (WIRB) with Institutional Review Board (IRB) Study Number 20170658 at the Institute for Systems Biology and 1178906 at Arivale (both in Seattle, WA). |

Note that full information on the approval of the study protocol must also be provided in the manuscript.
