# Supplementary material for: The geometry of clinical labs and wellness states from deeply phenotyped humans
Source: Nat Commun. 2021 Jun 11;12:3578. doi: 10.1038/s41467-021-23849-8 (PMC8196202; doi:10.1038/s41467-021-23849-8)
Supplement: Supplementary file 8 — Description of Additional Supplementary Files [file 41467_2021_23849_MOESM8_ESM.docx]

Title: Supplementary Data 1

Description: The table describes the 67 clinical labs that were used to construct the tetrahedron.

Title: Supplementary Data 2

Description: The full table of features and their P-values that were tested in the enrichment analysis. Every datatype is in a separate sheet and ordered from the smallest to the largest P-value, including all features - both significant and not significant (an index for the datatypes is available in the first sheet). The Bonferroni correction threshold is 3.9e-06.

Title: Supplementary Data 3

Description: This table summarizes the sensitivity of the enrichment analysis to bin size. To determine the sensitivity of the enrichment analysis to the bin size we ran the analysis with 15, 20, 25 and 40 bins. In general, when increasing the number of bins, fewer variables were found to be enriched with significant P-value after Bonferroni correction.

Title: Supplementary Data 4

Description: The table summarize all adverse events that were reported by the participants. There were 80 unique adverse events, and they are ordered from the most prevalent one (kidney stones - reported by 5 participants), to the least reported events (reported by only one participant, like liver cancer).

Title: Supplementary Data 5

Description: The table describes the Gender enrichment analysis next to every archetype. Next to archetype II that was enriched for females, 100% of the individuals in the first bin were females, and next to archetype III that was enriched for males, 97% of the individuals in the first bin were males.
